# Trimeric and Tetrameric Cationic Styryl Dyes as Novel Fluorescence and CD Probes for ds-DNA and ds-RNA

**DOI:** 10.3390/ijms25115724

**Published:** 2024-05-24

**Authors:** Dijana Pavlović Saftić, Ivona Krošl Knežević, Fernando de Lera Garrido, Juan Tolosa, Dragomira Majhen, Ivo Piantanida, Joaquín Calixto García Martínez

**Affiliations:** 1 Division of Organic Chemistry & Biochemistry, Ruđer Bošković Institute, 10000 Zagreb, Croatia; dijana.pavlovic.saftic@irb.hr (D.P.S.); ivona.krosl@irb.hr (I.K.K.); 2 Department of Inorganic and Organic Chemistry and Biochemistry, Faculty of Pharmacy, Universidad de Castilla-La Mancha, C/José María Sánchez Ibáñez s/n, 02008 Albacete, Spain; fernandoj.lera@gmail.com (F.d.L.G.); juan.tolosa@uclm.es (J.T.); 3 Regional Center for Biomedical Research (CRIB), Universidad de Castilla-La Mancha, C/Almansa 13, 02008 Albacete, Spain; 4 Division of Molecular Biology, Ruđer Bošković Institute, 10000 Zagreb, Croatia; dragomira.majhen@irb.hr

**Keywords:** styryl dyes, DNA binding, RNA binding, fluorescence, circular dichroism, cell non-toxicity

## Abstract

The wide use of mono- or bis-styryl fluorophores in biomedical applications prompted the presented design and study of a series of trimeric and tetrameric homo-analogues, styryl moieties arranged around a central aromatic core. The interactions with the most common biorelevant targets, ds-DNA and ds-RNA, were studied by a set of spectrophotometric methods (UV-VIS, fluorescence, circular dichroism, thermal denaturation). All studied dyes showed strong light absorption in the 350–420 nm range and strongly Stokes-shifted (+100–160 nm) emission with quantum yields (*Φ_f_*) up to 0.57, whereby the mentioned properties were finely tuned by the type of the terminal cationic substituent and number of styryl components (tetramers being red-shifted in respect to trimers). All studied dyes strongly interacted with ds-DNA and ds-RNA with 1–10 nM^−1^ affinity, with dye emission being strongly quenched. The tetrameric analogues did not show any particular selectivity between ds-DNA or ds-RNA due to large size and consequent partial, non-selective insertion into DNA/RNA grooves. However, smaller trimeric styryl series showed size-dependent selective stabilization of ds-DNA vs. ds-RNA against thermal denaturation and highly selective or even specific recognition of several particular ds-DNA or ds-RNA structures by induced circular dichroism (ICD) bands. The chiral (ICD) selectivity was controlled by the size of a terminal cationic substituent. All dyes entered efficiently live human cells with negligible cytotoxic activity. Further prospects in the transfer of ICD-based selectivity into fluorescence-chiral methods (FDCD and CPL) is proposed, along with the development of new analogues with red-shifted absorbance properties.

## 1. Introduction

Small molecules acting as antitumor drugs are extensively used in therapy, whereby most activity pathways are based on the non-covalent interactions of small molecules with DNA. Some such molecules (prodrugs) are not toxic by themselves and, thus, are harmlessly distributed in the patient’s body–but irradiation of particular body parts by light triggers various molecular mechanisms in prodrugs, causing strong toxicity and well-localized antitumor activity. Such an approach is called photodynamic therapy (PDT) and is intensively used in clinics on well-defined, non-operable tumors. Such photo-active molecules are also strongly fluorescent, often characterized by a long-living triplet state, allowing oxygen sensitization or similar processes [1].

Their fluorescence can also be used in bioimaging, and it is important in the viewing of individual organelles, organs, and organisms because it may give exceptional spatial and temporal resolution. This approach, when combined with the right set of imaging reagents, may be used to selectively monitor “targeted analytes” in cells, organs, and even complete organisms [2,3,4,5,6,7,8,9]. As a general rule, a good fluorescent probe should be able to target specific organelles while not influencing their shape and physiology [10,11,12,13,14].

Due to their favorable optical properties and the plethora of available synthetic approaches and modification protocols, styryl dyes provide an attractive scaffold for creating cellular probes. This building block can be found among several antiviral, antifungal [15,16], antibacterial [17], and anticancer medicines [18] and inhibitors of HIV integrase [19]. This class of compounds has attracted a lot of interest because of its electroluminescence and photochromic capabilities, which might make them useful in optical devices [20,21] or as sensors [22] and biosensors [23,24,25]. Further, a 4-aminostyryl derivative of 6-methylquinoline showed potential therapeutic and diagnostic characteristics against Alzheimer’s disease [26]. A styryl-quinoline dye with the dipicolyamine moiety exhibited multicolor fluorescence recognition of various metal ions [27]. Also, membrane dyes SynaptoGreenTM and SynaptoRedTM (formerly known as FM^®^ dyes) were used to track endocytic vesicles.

Recently, we reported on novel styryl and bis-styryl dyes [28], with particularly intriguing remarkable Stokes red shifts of emission maxima up to Δ*λ* = 150 nm. These dyes showed that the volume or positioning of terminal aromatic substituents directly controlled the DNA/RNA binding, shifting between DNA/RNA groove binding or DNA/RNA intercalation; consequently, some dyes yielded specific fluorimetric or CD responses for various DNA or RNA. Some dyes showed promising antiproliferative activity combined with selective fluorimetric intracellular staining (thus acting as theranostic agents), while others were biologically inactive but still selectively staining various cellular organelles [28,29]. Further, we very recently designed and studied monocationic styryl dyes [30] with fluorescence quantum yields strongly dependent on the selection of the *N*-quaternary heterocycle. New dyes interacted non-covalently with DNA/RNA by micromolar affinity, yielding a strong fluorescence increase strongly dependent on a heterocycle of a dye. Studied dyes showed mostly negligible cytotoxic activity, combined with localization between mitochondria and lysosomes, selectivity finely tuned by choice of the heterocycle. In this line, and as precursor results, we had shown that tris(styryl)benzenes functionalized properly with a peptide sequence able to bind to A/T-rich sites in DNA and were able to act as biomarkers for DNA three-way junctions [25].

**Scheme 1:**
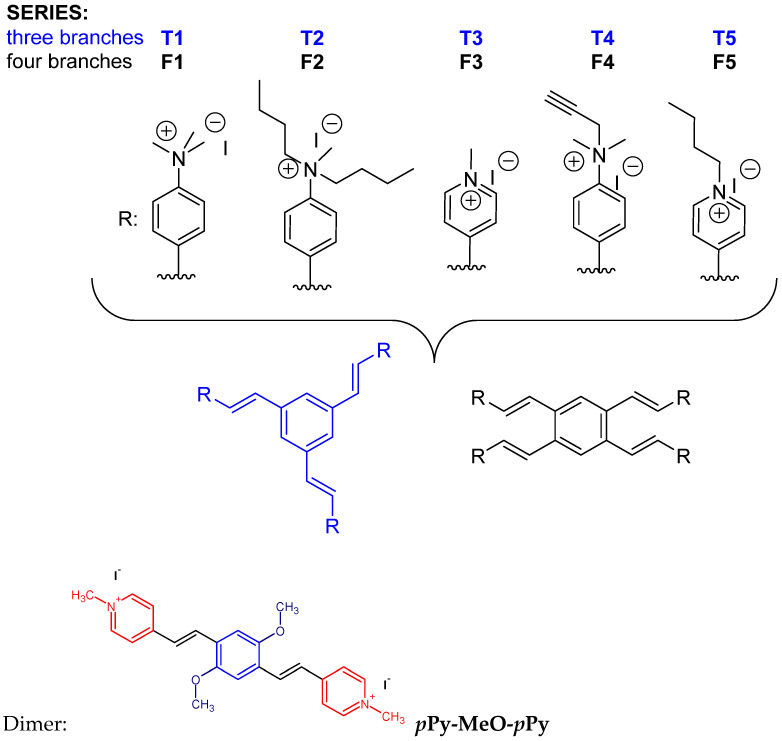
Structures of studied dyes: **T** represents trimeric core and **F** tetrameric core, to which are attached substituents R to form two groups of five analogues each. Dimer ***p*Py-MeO-*p*Py**: previously studied analogue of T3 and F3 [28].

Intrigued by the obtained results for mono- and bis-styryl dyes [28,30], the question arose whether the multiplicity of the styryl motif in one molecule could improve some aforementioned properties. Thus, we designed a novel generation of styryl dyes (Scheme 1) based on a variation of three distinct structural features:

(a) multiplicity of styryl motif through attaching three or four styryl moieties to one central aromatic core;
(b) type of positive charge by comparing heterocyclic cationic species with exocyclic per-alkylated amines;
(c) steric shielding of positive charge by the introduction of smaller or larger alkylating groups.

To make it easier for the reader to follow the discussion, structures with three branches have been named **T1** to **T5**, and those with four branches have been named **F1** to **F5**.

Polynucleotides we used to investigate modes of binding were the most commonly used ds-DNA representatives with specific structural features (Supp. Info., Table S1): *calf thymus* (ct)-DNA characterized by typical B-helical structure and approximately equimolar number of G–C and A–T base pairs, synthetic polynucleotides poly(dAdT)_2_, and poly(dGdC)_2_ also having B-helical structure, but the latter one characterized by guanine amino groups sterically blocking the minor groove, and a synthetic ds-RNA representative, poly A–poly U, characterized by A-helical structure [31,32].

## 2. Results and Discussion

### 2.1. Synthesis

**Scheme 2:**
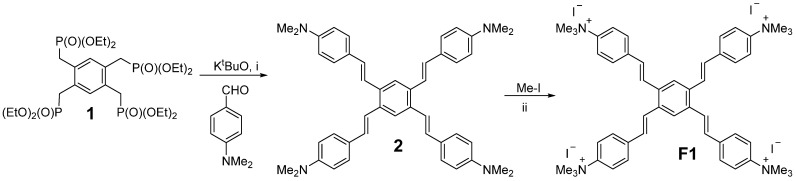
Synthesis of **F1** (i = 0 °C to rt, THF, 3 h. ii = rt, DCM, 48 h) as an example of the approach to all the molecules studied.

The preparation of the ten compounds tested as probes in this article involves the Horner–Wadsworth–Emmons reaction of the corresponding tris- or tetrakis-phosphonate derivative with three or four equivalents of the selected aldehyde in the presence of potassium *tert-*butoxide, followed by the full quaternization of the corresponding dialkylamine or pyridine moieties with an excess of the alkylating iodide. Scheme 2 shows the synthetic route to the preparation of **F1** as an example of the procedure. All ^1^H-NMR spectra of the products after the HWE reaction show a coupling constant of 16 Hz between the vinylic protons, proving the absence of any formation of *cis* adducts. Further details about experimental conditions and the characterization of these compounds are available in the Supp. Info.

### 2.2. Photophysical Studies of the New Dyes in Aqueous Solutions

**Figure 1:**
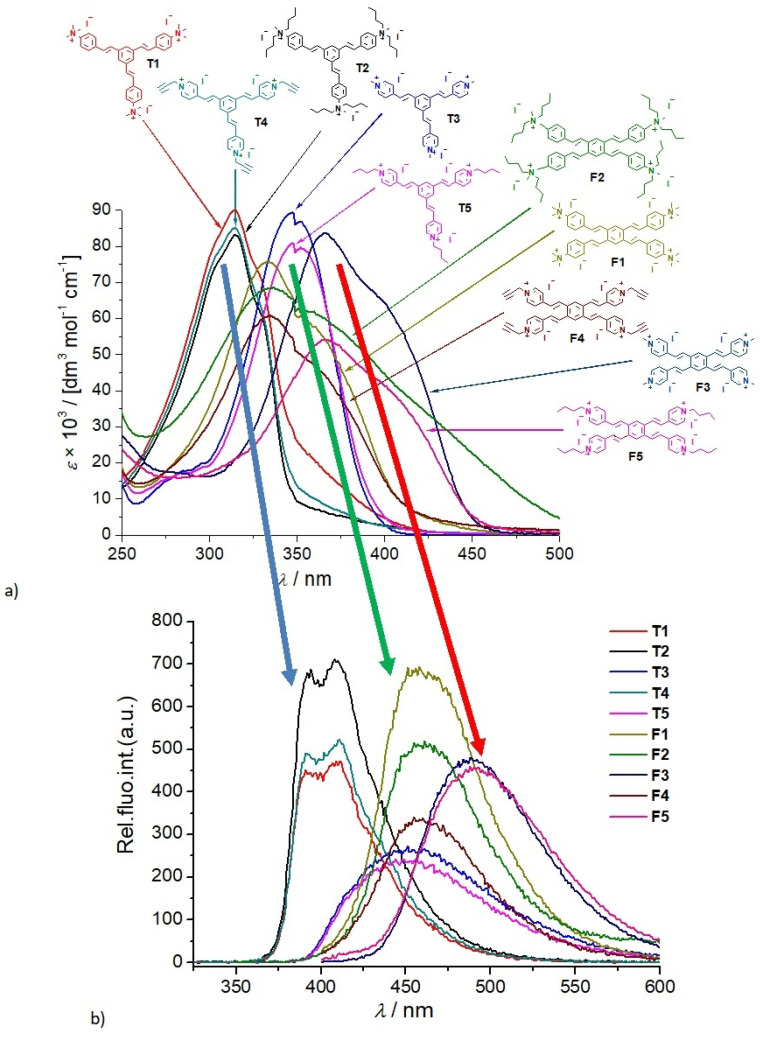
(**a**) Comparison of UV-Vis spectra of novel dyes. (**b**) Fluorescence spectra of the dyes at *c* = 2.5 × 10^−7^ M.

All compounds were dissolved in DMSO at 1 × 10^−2^ mol dm^−3^. The stock solutions were stored at +4 °C, and working aliquots were kept at +25 °C. No visible precipitation in working solutions was noticed. The UV-Vis spectra in aqueous solution (Figure 1a) are proportional to the concentration of dye up to *c* = 1 × 10^−5^ M (4 × 10^−6^ M) (Supp. Info., Figures S1a–S10a), yielding molar absorptivity (Table 1, *ε*). The obtained results do not support the formation of aggregates in the conditions used.

The UV-Vis spectra of all studied dyes were not significantly temperature-dependent (Supp. Info., Figures S1b–S10b), revealing excellent stability upon heating and reproducibility upon reversibly cooling down the sample solutions within the 25 °C to 95 °C temperature range. However, the UV-Vis spectra of all dye aqueous solutions changed when stored for several days in the dark, suggesting chemical decomposition, thus fresh solutions from DMSO stock solution were prepared daily.

Detailed analysis of UV-Vis spectra (Figure 1a) revealed a strong impact of the number of substituents on the central aryl, the tetra-substituted analogues generally being red-shifted in comparison to tri-substituted counterparts. This has been previously observed by some of us in analogous derivatives [21,33], and it is because a conjugation is more effective in the tetra derivatives, leading to a reduction in the energy gap between the HOMO and LUMO and thus a red shift of absorption. Also, methyl- and buthyl-pyridylium analogues (**T3**, **F3**, **T5**, and **F5**) cause pronounced bathochromic shift of the absorption maximum (Δ*λ* > +30 nm) in comparison with trialkyl-*N*-quaternary-substituted compounds **T1**, **T2**, **T4**, **F1**, **F2**, and **F4**. This is because the quaternization of pyridinic nitrogen leads to a more electron-withdrawing group than the corresponding anilinium derivative. Being more electron-withdrawing, it further destabilizes the HOMO and reduces the HOMO–LUMO energy gap, red-shifting the absorbed light.

All compounds showed fluorescence emission maxima following the trend observed for UV-Vis maxima (Figure 1a,b), namely tetra-substituted and pyridylium analogues being bathochromically shifted in comparison to tri-substituted and trialkyl-*N*-quaternary analogues. All studied dyes exhibit remarkable Stokes shifts, ranging from 97 nm to 165 nm, depending on the employed chromophores as building blocks. These high Stokes shifts have been observed previously and are due to a structural reorganization of the molecule in the excited state, leading to a planarization of one of its branches in the case of the tri-substituted and two branches in the case of the tetra-substituted. This planarization in the excited state leads to an increase in the conjugation of the molecule in the excited state, an energetic stabilization of this state, and a more red-shifted emission of light [21,33]. This feature gives them an additional advantage in terms of possible biological applications of the new probes [34,35]. The emission intensity of all dyes was proportional to concentration within the 5 × 10^−8^–2.5 × 10^−7^ M range (Supp. Info., Figures S11–S20), pointing to the absence of any dye aggregation.

The absolute fluorescence quantum yields (*Φ_f_*) gave very different values between 0.57 and 0.04 (Table 1). Previous observations of analogues of tri- and tetra-substituted oligo- and tetra-substituted oligostyryl benzenes show that, in most cases, *Φ_f_* ranges from moderate to good but are very dependent on the functional group that incorporates the positive charge [21,33], and this is what is observed in this case. Generally, the lowest *Φ_f_* was found for methyl-pyridylium analogues **T3** and **F3**, while the highest *Φ_f_* values were obtained for trialkyl-substituted exocyclic nitrogen analogues **T2** and **F1**.

Detailed analysis of time-correlated single-photon counting (TC-SPC) results (Table 1, τ) revealed that τ values are controlled by the terminal cationic moiety, exocyclic cations having significantly longer emission lifetime with respect to the pyridylium analogues. Intriguingly, tetrameric analogues in general had somewhat shorter τ values in comparison to trimer analogues, suggesting the facilitation of the radiative relaxation process by more densely packed styryl moieties.

Comparison of spectroscopic properties (Table 1) of **T3** and **F3** with previously studied dimer ***p*Py-MeO-*p*Py** [28] revealed comparable values of *ε* (if normalized to single Py-styryl unit) but also showed many intriguing differences. Namely, dimer UV-Vis and emission maxima are strongly red-shifted in comparison to **T3** and **F3,** and fluorescence quantum yield of the dimer is much higher, although emission lifetimes are of a similar order of magnitude. Thus, linking of three or four Py-styryl units through an aromatic center, which distributes aromatic conjugation over the complete system, actually blue-shifted their absorption and emission properties, which is less convenient for bioimaging applications.

### 2.3. Interactions of New Dyes with ds-DNA and ds-RNA

#### 2.3.1. Thermal Denaturation of ds-DNA/RNA

Thermal denaturation experiments provide information about the ds-polynucleotide helix thermal stability as a function of interaction with added small molecules [32]. The difference between the *T_m_* value of free ds-polynucleotide and a complex with a small molecule (Δ*T_m_* value) is an important factor in the characterization of small molecule/ds-polynucleotide interactions. For instance, moderate to strong stabilization (Δ*T_m_* > 5 °C) supports pronounced intercalative or minor groove binding interaction [36], whereas weak or negligible stabilization (Δ*T_m_* = 0–5 °C) suggests a binding process driven mostly by hydrophobic effect accompanied by weak H-bonding and/or electrostatic interactions–usually excluding classical intercalation as a binding mode.

The addition of studied dyes to ds-DNA or ds-RNA mostly resulted in moderate to strong stabilization of ds-helices (Table 2, Supp. Info., Figures S21–S24). Detailed analysis of results revealed general dependence of the thermal stabilization effect on the bulkiness of dye in general and, particularly, the terminal cationic substituent. Namely, insertion of a compound within the DNA/RNA groove has to be combined with efficient interaction of positive charges with the negatively charged DNA/RNA backbone, and smaller and more flexible trimers (T series) are sterically in advantage over bulky tetramers (F series). Particularly voluminous dibutyl-amine analogues have the lowest (**T2**) or even a negligible (**F2**) stabilization effect.

Since the tetrameric series showed poor differentiation between ds-DNA and ds-RNA, the trimeric series is discussed in more detail. The strongest selectivity in stabilization between ds-DNA and ds-RNA showed trimeric analogues: **T1** (two-fold selective toward ds-RNA) and oppositely **T5** (ten-fold selective toward ds-DNA). For these two compounds, selectivity can be related to the difference in the DNA and RNA binding sites–minor and major groove (Supp. Info., Table S1) [31,32], respectively. It seems that globular **T1** with three permethylated cations interacting with DNA-phosphate anions fits better in the more restrictive DNA minor groove than in the much deeper RNA major groove, corroborating well with the results of circular dichroism experiments (vide infra Figure 5 and related discussion). Oppositely, longitudinally extended pyridylium-buthyl cations of **T5** reverse selectivity in favor of the deep RNA major groove, in agreement with the results of circular dichroism experiments (vide infra Figure 4 and related discussion).

Comparison of tetramer **F3** data with a previously studied dimer [28] (Table 2) revealed a comparable stabilization effect if normalized per number of positive charges. However, trimer **T3** showed a significantly stronger stabilization effect, from both dimer and tetramer, which can be attributed to the optimized interplay between insertion of the hydrophobic part of the dye inside the DNA/RNA groove (likely leading by the central aromatic core) and three cationic arms staying out of the DNA/RNA groove and forming efficient electrostatic interactions with the anionic polynucleotide backbone.

#### 2.3.2. Spectrophotometric Titrations of the New Dyes with Polynucleotides

The strong emission and favorable Stokes shift of emission maxima allowed the fluorimetric titrations of investigated dyes with ds-DNA and ds- RNA at very low (50 nM) concentrations. The addition of any ds-DNA or ds-RNA to studied dyes resulted in a strong quenching of their emission (Figure 2, Supp. Info, Figures S25–S44), whereby none of the dyes showed a particular selectivity response toward DNA or RNA. The titration data are processed by a non-linear fitting procedure to the Scatchard equation [37,38,39], yielding binding constants (Figure 2c, Supp. Info, Figures S25–S44, Table 3).

Generally, binding constants obtained from fluorimetric titrations were similar and remarkably high (log *K*_s_ ≥ 8–9), with studied dyes showing no significant selectivity between ds-DNA and ds-RNA. The exception is **F3**, which shows an order of magnitude preference for ds-RNA, likely due to the optimized insertion into the deep and narrow RNA major groove in comparison to the less deep and broader DNA minor groove (see Supp. Info., Table S1: Structural properties of studied ds-DNA and ds-RNA). A previously studied dimer [28] showed lower binding constants with respect to **T3** and similar to **F3**, in agreement with thermal denaturation data (Table 2). Thus, binding affinity and thermal stabilization effects are controlled by fine interplay between the flexibility/bulkiness of dye (controlling insertion into DNA/RNA groove) and number of positive charges, which are able to interact electrostatically with the polynucleotide backbone.

#### 2.3.3. Circular Dichroism (CD) Experiments

To gain insight into the structural properties of dye/polynucleotide complexes, we have chosen circular dichroism (CD) spectroscopy as a highly sensitive method toward chirally related conformational changes in the secondary structure of polynucleotides [40]. In addition, achiral small molecules can eventually acquire an induced CD spectrum (ICD) upon binding to polynucleotides, which could give useful information about modes of interaction [41,42]. It should be noted that studied compounds are achiral and therefore do not possess an intrinsic CD spectrum.

The addition of any of the studied compounds resulted in strong changes in the 230–295 nm range, attributed to the bands of ds-DNA or d-RNA. Such changes in the chiral helicity of DNA/RNA are in line with strong binding constants determined by fluorimetric titrations (Table 3) and, also, are in agreement with thermal stabilization effects (Table 2).

Furthermore, many dyes upon binding to ds-DNA/RNA exhibited pronounced induced (ICD) bands (Supp. Info., Figures S45–S48) as the consequence of uniform orientation of dye chromophores with respect to the polynucleotide chiral axis. For all dyes, the shape and sign of ICD bands were strongly dependent on the ratio *r* = [dye]/[polynucleotide], whereby bands observed for r ≤ 0.1 are mostly weak, single-sign bands, which could be attributed to single molecules bound in one dominant binding site: ds-DNA minor groove or ds-RNA major groove [41]. However, at a larger excess of dye (r > 0.1), dye aggregates are formed along a polynucleotide, inducing, in many cases, specific pronounced bisignate excitons-coupled bands for a particular target [41].

In general, trimeric dyes showed much stronger ICD bands in comparison to their tetrameric analogues, which could be attributed to the smaller size of trimeric series and consequent deeper molecule immersion within DNA/RNA grooves, resulting in the more uniform orientation of dye chromophores with respect to the DNA/RNA chiral axis. For that reason, some trimeric dyes showed highly selective or even specific ICD bands for certain types of DNA or RNA secondary structures.

**Figure 3:**
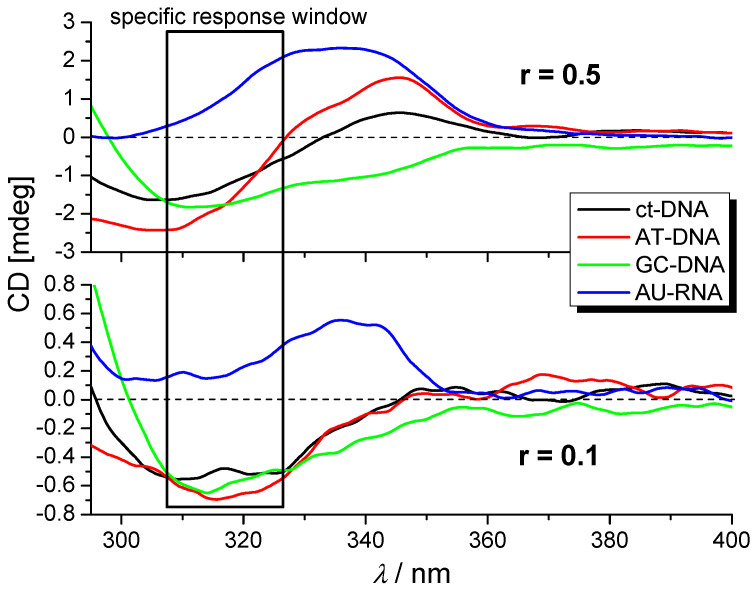
CD titration of various ds-DNA and ds-RNA with **T2** at ratio *r*_[dye]/[polynucleotide]_ = (DOWN) 0.1 and (UP) 0.5. Note the square that marks the response window (*λ* = 308–324 nm) specific for AU-RNA. Performed at pH 7, buffer sodium cacodylate, *I* = 0.05 M.

For instance, the complex of the most voluminous trimeric analogue **T2** with all studied ds-DNA at all ratios gave negative ICD bands within the 308–324 nm range, at variance to the **T2**/AU-RNA complex yielding a positive ICD band (Figure 3). Such a specific ICD signal can be correlated to the very deep RNA major groove (Supp. Info., Table S1), which differently oriented the **T2**-chromophore in comparison to the much shallower ds-DNA minor grooves.

Other, smaller trimeric dyes show weak, non-selective ICD bands for a single molecule bound to DNA/RNA; however, at an excess of dye over DNA/RNA (ratio r > 0.1), several molecules aggregate within the binding site, yielding, in some cases, specific and very strong ICD bands.

**Figure 4:**
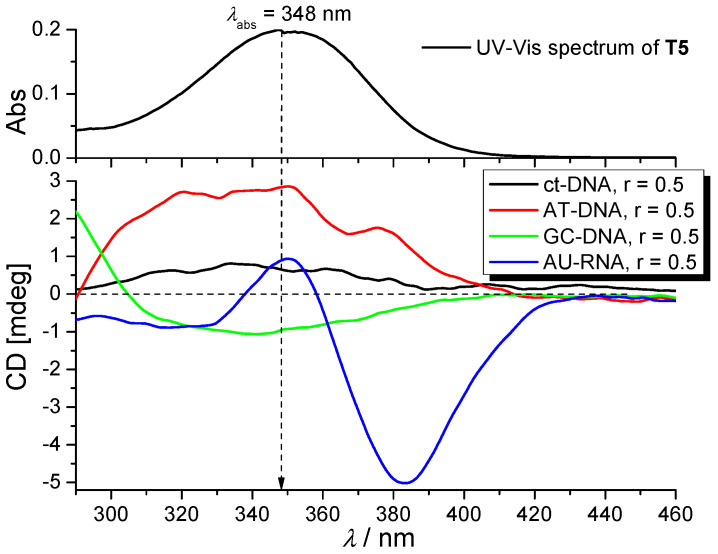
CD titration of various ds-DNA and ds-RNA with **T5** at ratio *r*_[dye]/[polynucleotide]_ = 0.5. Note a strong negative band at 383 nm specific for AU-RNA. Performed at pH 7, buffer sodium cacodylate, *I* = 0.05 M.

For instance, **T5** (a smaller analogue of **T2**, having a single butyl attached to a pyridylium chromophore) aggregates in a very specific manner within the very deep AU-RNA major groove (Supp. Info., Table S1), forming the exciton-coupled bisignate bands strongly bathochromically shifted (+30 nm) with respect to **T5**/ds-DNA complexes (Figure 4). This structural selectivity can also be correlated to the thermal stabilization selectivity toward ds-RNA, as noted in the discussion related to Table 2 (vide supra).

**Figure 5:**
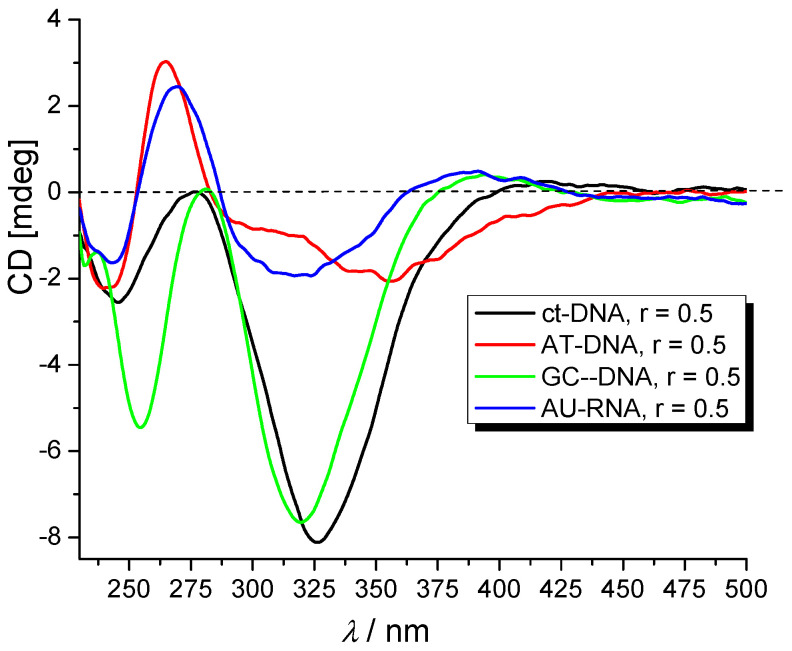
CD titration of various ds-DNA and ds-RNA with **T1** at ratio *r*_[dye]/[polynucleotide]_ = 0.5. Performed at pH 7, buffer sodium cacodylate, *I* = 0.05 M.

Further, at aggregation conditions for **T1**, the smaller analogue of **T2** showed a much stronger negative ICD band at *λ* = 325 nm for GC-containing DNAs in comparison to AT-DNA or AU-RNA (Figure 5). Such a highly selective response can be correlated to the specific property of the GC-DNA minor groove, within which amino groups of guanin sterically reduce the orientation of **T1** chromophores, forcing them into orientation with respect to the DNA chiral axis into the optimal position for ICD response. The AT-DNA or AU-RNA grooves are free of steric hindrances and allow variation of chromophore orientations, diminishing the intensity of ICD bands. Moreover, this structural selectivity can be correlated to the thermal stabilization selectivity of **T1** toward ct-DNA over AU-RNA (vide supra discussion to Table 2), whereby insertion of **T1** into the deep major groove of AU-RNA partially hampers electrostatic interactions of cations with polynucleotide phosphate anions, thus resulting in a diminished stabilization effect.

### 2.4. Biological Experiments

**Figure 6:**
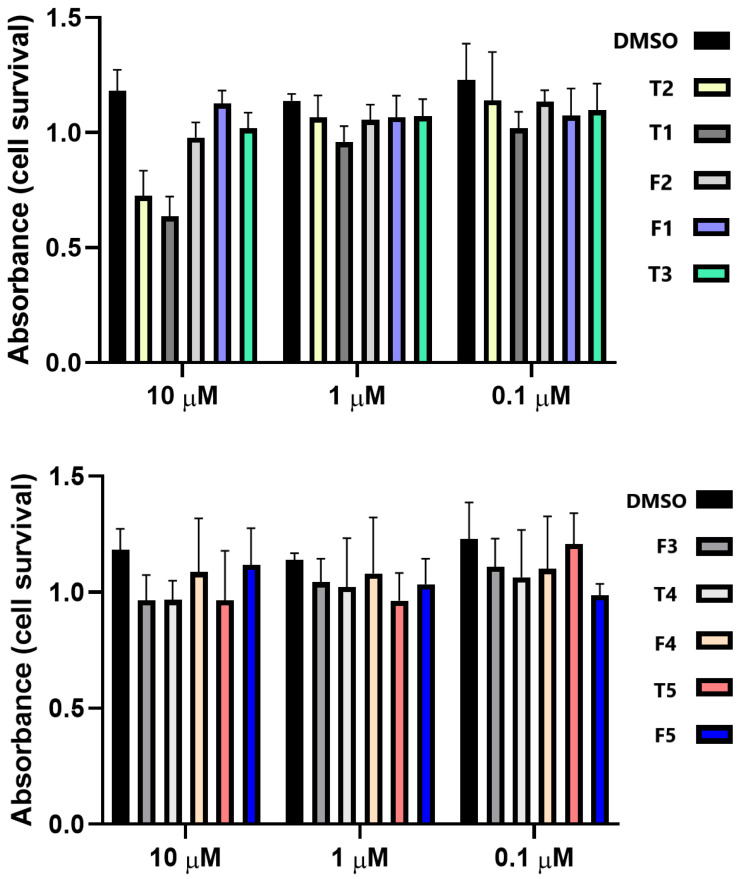
Cell survival of A549 cells treated with different concentrations of all studied dyes. Data are presented as mean ± SD made in four replicates, relative to the control samples. Control samples are cells treated with DMSO at the same concentration as a tested compound. Representative data from three independent experiments, which yielded similar results, are shown.

Prior experiments in this work showed that studied dyes strongly bind to ds-DNA/RNA, which can be the cause of strong cytotoxicity [43]. Thus, we studied the impact of **dye** addition to the human lung carcinoma cell line (A549) by MTT assay (Figure 6). Compared to the control cells (cells treated with DMSO, which was used as a diluent for the dyes), cells treated with the studied compounds exhibited a negligible decrease in cell survival even at the highest tested concentration (10 μM), except **T1** and **T2**, showing minor activity at the highest concentration.

#### Confocal Microscopy

The observed negligible cytotoxicity of dyes could be a consequence of the inability of dyes to enter living cells, as known for propidium iodide or other fluorescent dyes commonly used to test the viability of cells. To verify successful cellular uptake, we treated A549 cells with 10 μM concentration of dyes for 90 min and performed live cell confocal microscopy. For this study, we have chosen trimeric derivative **T1** because of its minor toxicity (likely caused by efficient cellular uptake) and tetrameric analogues **F3** and **F5** as representatives of non-toxic dyes with the most bathochromically shifted excitation and emission spectra particularly.

**Figure 7:**
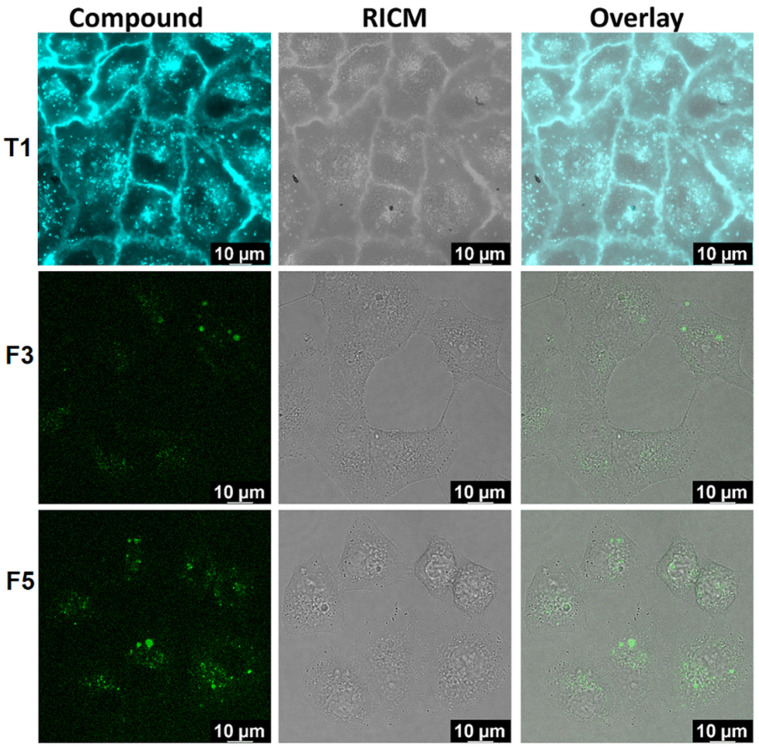
A549 cells were treated with 10 µM of the respective compound for 90 min at 37 °C and subsequently observed by confocal microscopy. Intracellular distribution of **T1** (*λ*_exc_ = 350 nm; *λ*_em_ = 400–500 nm)**, F3** (*λ*_exc_ = 405 nm; *λ*_em_ = 450–550 nm), and **F5** (*λ*_exc_ = 405 nm; *λ*_em_ = 450–550 nm) was observed.

As can be seen in Figure 7 (TOP), compound **T1** is localized in the cell membrane, indicating this dye has a preference for a phospholipid bilayer. Due to cell auto-fluorescence in the used spectral range and weak emission of **F3** and **F5**, we could not perform co-localization experiments with specific markers for cytoplasmic organelles; however, it can be seen that those compounds enter the cell (Figure 7, center, bottom).

The obtained results show that the studied compounds, **T1**, **F3,** and **F5,** successfully enter the cells. Due to the specific localization to the cell membrane and satisfactory fluorescence properties, **T1** merits further assessment as a potential marker for the lipid bilayer.

Whenever several different dyes have to be simultaneously employed in living cells, they have to differ by emitting properties sufficiently. In this respect, only a few membrane-selective blue dyes for living cells are commercially available. For instance, BioTracker™ 400 Blue Cytoplasmic Membrane Dye (Fort Lauderdale, FL, USA, Excitation: 366 nm; Emission: 441 nm) is characterized by Stokes shift of +80 nm, whereas the presented dyes show Stokes shift up to +160 nm, which allows much better differentiation from other simultaneously employed dyes.

## 3. Materials and Methods

### 3.1. General

In air- and moisture-sensitive reactions, all glassware was flame-dried and cooled under nitrogen. All reagents and solvents obtained from commercial sources (Merck Life Science S.L.U., Madrid, Spain) were used as received, except for tetrahydrofuran, which was freshly distilled over sodium/benzophenone ketyl under a positive pressure of dry nitrogen. ^1^H NMR and ^13^C NMR spectra were acquired at room temperature on a Varian Inova-500 instrument (Palo Alto, CA, USA). The NMR chemical shifts (δ) are given in ppm and are referenced to the residual protons of the deuterated solvent or carbon nuclei of CDCl_3_ (^1^H, δ = 7.27 ppm; ^13^C, δ = 77.0 ppm) or DMSO-*d6* (^1^H, δ = 2.50 ppm; ^13^C, δ = 39.52 ppm). Acidic impurities in CDCl_3_ were removed by treatment with anhydrous K_2_CO_3._

### 3.2. Synthesis

Triphosphonate and tetraphosphonate were obtained by the Arbuzov reaction of 1,3,5-tris(bromomethyl)benzene and 1,2,4,5-tetra(bromomethyl)benzene with triethyl phosphite following a standard methodology [44]. As an example, the synthesis of **F1** is described in Scheme 2.

**Compound 2**. Tetraphosphonate (212 mg, 0.312 mmol) and 4-dimethylamino benzaldehyde (187 mg, 1.25 mmol) were dissolved in dried tetrahydrofuran (10 mL) in a two-necked flask (25 mL) under argon atmosphere. The solution was cooled in an ice bath, and then ^t^BuOK (186.86 mg, 1.665 mmol) was added very slowly. Then, the reaction was allowed to reach room temperature and was stirred for 3 h. Water was added, and the yellow precipitate formed was filtered off. The isolated compound was purified by boiling it in hot methanol. Yield: 92%. ^1^H NMR (CDCl3, 500 MHz): δ 3.00 (s, 24H, 8 × CH3), 6.75 (A of ABq, 8H, J = 8.5 Hz, ArH), 7.03 (A of AB, 4H, J = 16.0 Hz, 4 × CH=), 7.30 (B of AB, 4H, J = 16.0 Hz, 4 × CH=), 7.48 (B of ABq, 8H, J = 8.5 Hz, ArH), 7.76 (s, 2H, ArH). ^13^C RMN and DEPT (CDCl3, 125 MHz): δ 150.1 (C), 135.1 (C), 130.4 (CH), 127.7 (CH), 126.4 (C), 123.8 (CH), 122.6 (CH), 112.5 (CH), 40.5 (CH3). MALDI-TOF MS (dithranol): *m/z* 658.8 [M]+•.

**Compound F1**. Compound **2** (100 mg, 0.204 mmol) was dissolved in dichloromethane and an excess of iodomethane (284 mg, 2 mmol) was added. The reaction mixture was stirred for 3 days. Compound **F1** precipitated in the reaction medium. It was filtered and washed with cold dichloromethane (yellow powder, 90%). ^1^H NMR (DMSO-d6, 500 MHz): δ 3.66 (s, 36H, 12 × CH3), 7.52 (d, 4H, J = 16.5 Hz, 4 × CH=), 7.94 (d, 4H, J = 16.5 Hz, 4 × CH=), 8.01 (d, 8H, J = 7.0 Hz, ArH), 8.04 (d, 8H, J = 7.0 Hz, ArH). 8.18 (s, 4H, ArH). ^13^C RMN y DEPT (DMSO-d6, 125 MHz): δ 146.2 (C), 138.9 (C), 135.2 (C), 129.4 (CH), 128.1 (CH), 127.7 (CH), 124.0 (CH), 120.8 (CH), 56.4 (CH3). MALDI-TOF MS (dithranol): *m/z* 673.8 [M–4I–3CH3]+•.

### 3.3. Spectrophotometric Characterization

Absorption spectra were recorded on a Varian Cary 100 Bio spectrophotometer (Palo Alto, CA, USA). Fluorescence measurements were performed on a Varian Cary Eclipse fluorimeter (Palo Alto, CA, USA), taking care that the absorbance of a sample at the excitation wavelength was >0.05 to avoid any impact of the inner filter effect. Fluorescence decays were measured by time-correlated single-photon counting (TC-SPC) on an Edinburgh FS5 spectrometer (Edinburgh Instruments Ltd., Livingston, UK) in a previously degassed aqueous solution of dye. Absolute quantum yields of fluorescence (*Φ*_f_) were measured by the Integrating sphere SC-30 (Edinburgh FS5 Inst.). CD spectra were recorded on a JASCO J815 spectropolarimeter (JASCO, Easton, MD, USA). All spectrophotometric measurements were performed using an appropriate quartz cuvette of 10 mm path length in aqueous buffer solution (sodium cacodylate buffer, pH = 7, *I* = 0.05 M) at room temperature (25 °C).

### 3.4. Study of DNA/RNA Interactions

Polynucleotides were purchased as noted: poly dAdT–poly dAdT, poly dGdC–poly dGdC, poly A–poly U (Sigma Aldrich, St. Louis, MO, USA), *calf thymus* (ct) DNA (Sigma Aldrich, USA) and dissolved in sodium cacodylate buffer, *I* = 0.05 M, pH = 7.0. The ct-DNA was additionally sonicated and filtered through a 0.45 mm filter to obtain mostly short (ca. 100 base pairs) rod-like B-helical DNA fragments. Polynucleotide concentration was determined spectroscopically according to producer data as the concentration of phosphates (corresponds to *c*(nucleobase)).

Fluorescence titration experiments were performed by adding portions of the polynucleotide stock solution into the solution of the studied dye. CD experiments were performed by adding portions of the compound stock solution into the solution of the polynucleotide (*c* = 2 × 10^–5^ M) and were recorded with a scanning speed of 200 nm/min (an average of three accumulations). In CD experiments, a buffer background was subtracted from each spectrum. Every reported spectrophotometric titration was the average of at least two measurements.

Thermal denaturation curves for ds-DNA, ds-RNA, and their complexes with studied compounds were determined as previously described [45] by following the absorption change at 260 nm as a function of temperature. The absorbance of the ligands was subtracted from every curve, and the absorbance scale was normalized. *T_m_* values are the midpoints of the transition curves determined from the maximum of the first derivative and checked graphically by the tangent method. The Δ*T_m_* values were calculated by subtracting the *T_m_* of the free nucleic acid from the *T_m_* of the complex. Every Δ*T_m_* value here reported was the average of at least two measurements. The error in Δ*T_m_* is ±0.5 °C.

### 3.5. Biology

*Cells*. A549 (human lung carcinoma; ATCC CCL-185) were obtained from the ATCC Cell Biology Collection and were cultured according to the manufacturer’s instructions. Cells were grown in Dulbecco Modified Eagle’s Medium (DMEM, Sigma Aldrich, USA) supplemented with 10% of fetal bovine serum (FBS, Sigma Aldrich, USA) at 37 °C and 5% CO_2_ in a humidified atmosphere. Three biological replicas were performed for all experiments.

*Cytotoxicity assay–±MTT* [46]. Studied compounds were dissolved in an appropriate volume of dimethyl sulfoxide (DMSO) under sterile conditions to obtain 10 mM stock solutions and were kept in the dark at +4 °C. Before each assay, fresh working solutions were prepared from the stock solution by diluting them with DMEM. Cells were seeded on 96-well plates at concentrations of 7 × 10^3^ cells/well in 100 μL of DMEM (10% FBS) and left in the incubator overnight (37 °C, 5% CO_2_). The next day, 100 μL of the working solution was added to the wells, thus the final volume was 200 μL/well. All measurements were made in quadruplicate. Cells treated with the same dilutions of DMSO represented the control, while cells treated only with DMEM (10% FBS) represented the negative control. The plate was then incubated for the next 72 h (37 °C, 5% CO_2_). After incubation, the medium was removed, and 40 μL of an MTT solution was added to each well. The plate was incubated in the cell incubator for 3 h, allowing formazan crystals to form. After 3 h, 170 μL of DMSO was added to each well, and the plate was placed on a shaker for 20 min, allowing the crystals to dissolve. The absorbance of the MTT–formazan product was measured with a microplate reader at 600 nm, and the absorbance value correlates directly with cell survival.

Live cell imaging: Live imaging of the cells treated with compounds was performed on the A549 cell line (human lung carcinoma; ATCC CCL-185). Cells were seeded in Ibidi imaging cell chambers (Ibidi^®^, Gräfelfing, Germany) in 500 μL of the medium, with the concentration of 5 × 10^4^ cells/well, and left in the cell incubator for 48 h (37 °C, 5% CO_2_). After two days, cells were treated with 10 μM solution of a tested compound and left in the cell incubator for 90 min to allow the compound to enter the cells. After incubation, the medium was replaced with 500 μL of fresh DMEM, and cells were immediately observed by a Leica SP8 X confocal microscope (Leica Microsystems, Wetzlar/Mannheim, Germany).

## 4. Conclusions

Here, we described the synthesis of a novel series of tri- and tetrameric styryl dyes and fully characterized their spectroscopic and photochemical properties in biorelevant aqueous solution. The multiplicity of the styryl motif had a pronounced effect on the UV-Vis spectra, those of tetra-substituted analogues generally being slightly red-shifted in comparison to tri-substituted counterparts. Also, the type of positively charged moiety influenced strongly absorbance properties; the UV-Vis spectra of pyridylium analogues (**T3**, **F3**, **T5**, and **F5**) being strongly red-shifted (Δ*λ* > +30 nm) in comparison with trialkyl-*N*-quaternary-substituted compounds (**T1**, **T2**, **F1**, and **F2**). All compounds showed strong fluorescence, exhibiting remarkable Stokes shifts ranging from 97 nm to 165 nm, and the emission maxima agreed with the trend observed for UV-Vis maxima. Intriguingly, absolute fluorescence quantum yields (*Φ_f_*) were strongly dependent on a chromophore bearing positive charge, the pyridylium analogues showing the lowest and the exocyclic nitrogen analogues the highest Φ*_f_* values.

The thermal denaturation of ds-DNA or ds-RNA was more influenced (stabilized) by smaller trimeric compounds, likely due to their flexibility in adopting optimal positioning of cationic parts against the polyanionic DNA/RNA backbone. However, this molecule size-related selectivity was not evident from fluorimetric titrations, since the emission of all compounds was quenched similarly and the binding affinity was large (within the 1–10 nM^−1^ range) and similar. Thus, binding affinity and thermal stabilization effects are controlled by fine interplay between flexibility/bulkiness of dye (controlling insertion into the DNA/RNA groove, see Supp. Info. Table S1) and number of positive charges, which are able to interact electrostatically with the polynucleotide backbone.

Most intriguingly, induced circular dichroism (ICD) bands of trimeric (**T-series**) derivatives at a high ratio r = [dye]/[polynucleotide] were highly selective or even specific for a particular DNA or RNA secondary structure, depending on a dye size: the most voluminous trimeric analogue **T2** can efficiently differentiate between ds-DNA (minor groove, Supp. Info., see Table S1) and ds-RNA (major groove) by a negative/positive ICD band sign, a whereas its smaller analogue **T1** gave a strongly selective ICD band for GC-DNA with respect to AT-DNA or AU-RNA and the more planar pyridinium analogue **T5** yielded a specific ICD band for ds-RNA (major groove, Supp. Info., see Table S1). The tetrameric series gave much weaker ICD bands because their large size prevented deep immersion within DNA/RNA grooves and adequate orientation of chromophores with respect to the DNA/RNA chiral axis.

Further, studied dyes were negligibly toxic against human cells but efficiently entered cells within 1–2 h even at low (10 µM) concentrations. The confocal microscopy results show accumulation in cytoplasm organelles.

The presented results adequately addressed the questions instigating this research and strongly support further optimization of trimeric series of compounds in line with shifting the new dyes’ light absorption bathochromically into tissue-penetrating wavelengths (>500 nm). The comparison of spectroscopic properties of the presented dyes with their dimeric analogue [28] revealed that linking of three or four Py-styryl units through an aromatic center distributes aromatic conjugation over the complete system, causing blue-shifted absorption and emission properties. Therefore, oligomeric Py-styryl dyes should be preferably prepared by linking corresponding dimers by non-conjugated linkers, thus maintaining strongly red-shifted spectroscopic properties of the dimer, more convenient for bioimaging applications. Also, the application of the shown fine-tuned induced chiral recognition (ICD bands) of the DNA/RNA structure could be improved using fluorescence detected circular dichroism (FDCD) [47] and circularly polarized luminescence (CPL) [48] for an increase in the recognition sensitivity.

## Figures and Tables

**Figure 2:**
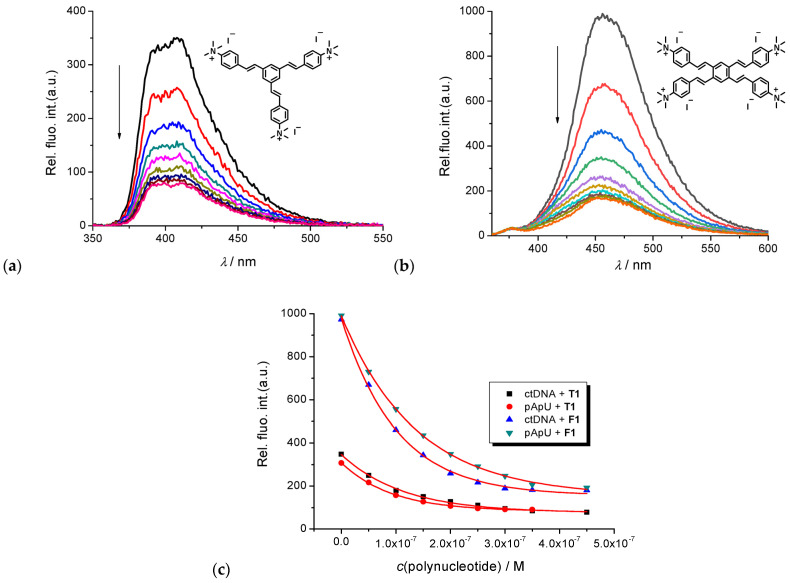
(**a**) Changes in fluorescence spectrum of tri-substituted dye **T1** (*c* = 5 × 10^−8^ M) upon titration with ctDNA; (**b**) changes in fluorescence spectrum of tetra-substituted dye **F1** (*c* = 5 × 10^−8^ M) upon titration with ctDNA; (**c**) changes in fluorescence intensity of **T1** and **F1** (*c* = 5 × 10^−8^ M) upon addition of polynucleotides at pH 7.0.

**Table 1:** Spectrophotometric data of the dyes in aqueous solution at pH 7.0, sodium cacodylate buffer, *I* = 0.05 M.

Compd.	^max^*λ*_abs_/[nm] (*ε* × 10^3^/[M^−1^ cm^−1^])	^b^ *Φ*_f_	^a^ *λ*_exc_ [nm]	*λ*_em_ [nm]	^a^ *τ* [ns]	*χ* ^2^
**T1**	314 (91.5)	0.27 ± 0.05	340	412	10.68 (14.04%) 20.33 (85.96%)	0.960
**T2**	315 (80.0)	0.41 ± 0.05	340	480	13.05 (33.74%) 21.50 (66.26%)	0.918
**T3**	348 (88.8)	0.04 ± 0.05	340	454	0.78 (90.42%) 5.03 (9.58%)	1.055
**T4**	314 (86.3)	0.37 ± 0.05	340	411	10.67 (18.28%) 20.24 (81.72%)	0.931
**T5**	348 (79.5)	0.29 ± 0.05	340	448	0.69 (98.06%) 4.55 (1.94%)	1.062
**F1**	333 (75.4)	0.57 ± 0.05	340	460	2.89 (21.82%) 7.03 (78.18%)	0.941
**F2**	335 (66.5)	0.20 ± 0.05	340	460	2.55 (20.77%) 6.69 (79.23%)	1.050
**F3**	366 (83.0)	0.07 ± 0.05	340	485	0.64 (59.91%) 3.56 (40.09%)	0.963
**F4**	334 (60.6)	0.35 ± 0.05	340	460	2.60 (28.08%) 6.58 (71.92%)	0.985
**F5**	366 (53.8)	0.06 ± 0.05	340	485	0.68 (55.54%) 3.63 (44.46%)	0.906
**^c^ dimer**	446 (35.0)	0.21 ± 0.05	457	605	0.19 (21%) 1.60 (79%)	0.98 < *χ*^2^ < 1.02

^a^ Samples were excited by pulsing diode EPLED340 at 340.1 nm. Emission was monitored at 460 and 500 nm. Aqueous solutions were purged by argon. The measurements were performed three times, and the average values are reported. The associated errors correspond to the maximum absolute deviation. ^b^ Absolute fluorescence quantum yields were measured at room temperature (25 °C) by the integrating sphere SC-30, Edinburgh Inst., for argon purged solutions. ^c^ Dimer ***p*Py-MeO-*p*Py**: previously studied analogue of **T3** and **F3** [28].

**Table 2:** Δ*T_m_* values ^a^ (°C) for ds-DNA or ds-RNA upon addition of dyes at ratio *r*_[dye]/[polynucleotide]_ = 0.1. Performed at pH 7, buffer sodium cacodylate, *I* = 0.05 M.

Compd.	ctDNA	pApU
**T1**	+11.9	+23.2
**T2**	+6.2	+5.1
**T3**	>+15.0	+8.5
**T4**	+12.0	+18.1
**T5**	+14.3	+1.5
**F1**	>+15.0	+12.5
**F2**	0	0
**F3**	+6.6	+6.4
**F4**	+4.8	+10.4
**F5**	+3.2	+1.7
**^b^ dimer**	+3.1	+1.8

^a^ Error in Δ*T_m_*: ±0.5 °C. ^b^ Dimer ***p*Py-MeO-*p*Py**: [28]; previously studied analogue of **T3** and **F3**.

**Table 3:** Binding constants ^a^ log *K_s_* and emission quenching efficiency (^b^ Δ*Int*) of complexes of **dyes** with ds-polynucleotides calculated by processing fluorimetric titrations at pH = 7.0, sodium cacodylate buffer, *I* = 0.05 M.

Compd.	ctDNA	pApU
**T1**	8.6 (0.23)	8.9 (0.30)
**T2**	8.6 (0.04)	8.9 (0.16)
**T3**	8.2 (0.24)	8.7 (0.13)
**T4**	8.9 (0.10)	8.6 (0.35)
**T5**	8.9 (0.10)	8.7 (0.14)
**F1**	9.3 (0.17)	8.6 (0.17)
**F2**	8.0 (0.09)	8.6 (0.08)
**F3**	7.1 (0.11)	8.2 (0.28)
**F4**	8.6 (0.17)	8.9 (0.17)
**F5**	8.9 (0.30)	7.6 (0.26)
**^c^ dimer**	6.9 (0.80)	7.5 (0.5)

^a^ Processing of titration data by means of the Scatchard equation [37,38,39]. For easier comparison, all log *K*_s_ values were re-calculated for fixed n[bound dye]/[polynucleotide] = 0.2. The correlation coefficients were >0.99 for all calculated *K*_s_. ^b^ ΔInt = Int_(100% complex)_/Int_0_. ^c^ Dimer ***p*Py-MeO-*p*Py**: [28]; previously studied analogue of **T3** and **F3**.

## Data Availability

All data are contained within this manuscript and the Supplementary Materials file.

## Supplementary Materials

The following supporting information can be downloaded at: https://www.mdpi.com/article/10.3390/ijms25115724/s1.
