# Supplementary material for: Trimeric and Tetrameric Cationic Styryl Dyes as Novel Fluorescence and CD Probes for ds-DNA and ds-RNA
Source: Int J Mol Sci. 2024 May 24;25(11):5724. doi: 10.3390/ijms25115724 (PMC11171523; doi:10.3390/ijms25115724)
Supplement: Supplementary file 1 [file ijms-25-05724-s001.zip › ijms-2980917-supplementary.pdf]

Supplementary Information

Tripodal and quadrupodal cationic styryl dyes as novel fluorescence and CD probes for ds-DNA and ds-RNA

1. **Physico-chemical properties**

1.1. Solubility

1.2. UV/Vis spectra, stability

1.3. Fluorescence spectra

2. **Study of interactions with double-stranded and single-stranded DNA/RNA**

2.1. Thermal denaturation experiments

2.2. Fluorescence titration experiments

2.3. Circular dichroism (CD) experiments

3. **Synthesis and characterization**

1. Physico-chemical properties

1.1. Solubility

All compounds (**T1-T5**, **F1**, and **F3-F5)** were dissolved in DMSO (c = 1 × 10^{-2} M). Compound **F2** is less soluble in DMSO. The highest stock solution obtained in DMSO was c = 7 × 10^{-4} M.

1.2. UV/Vis spectra, temperature stability

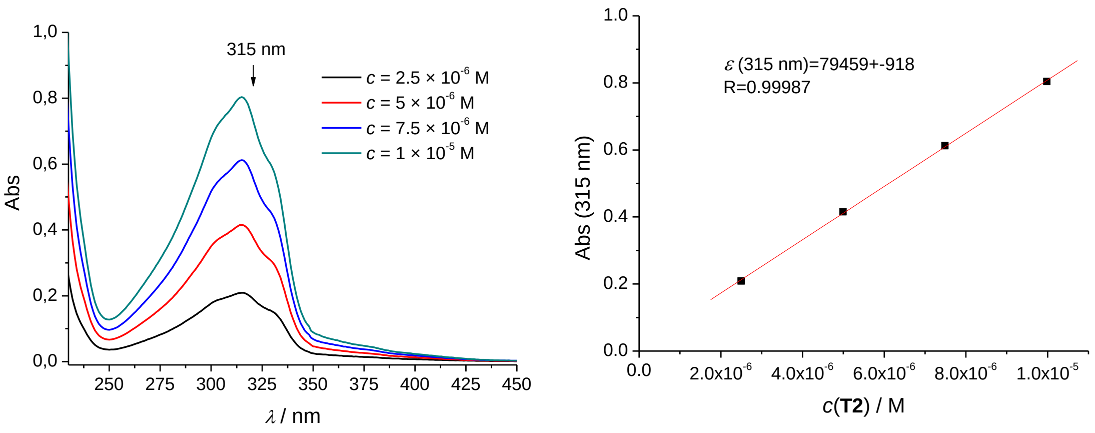

a

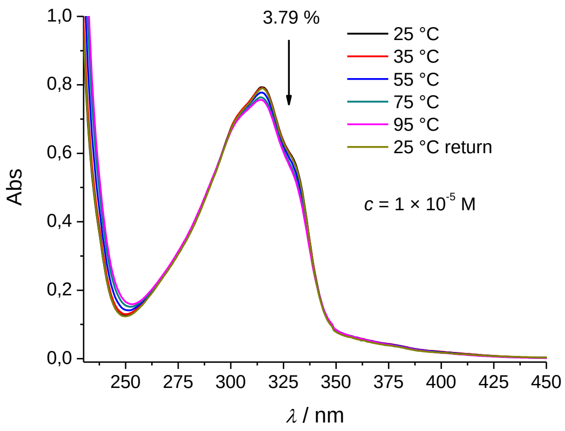

b

Figure S1. UV/Vis spectra changes of **T2**: a) Concentration dependence (concentration range 2.5 × 10^{-6} – 1 × 10^{-5} M), and b) Temperature dependence (temperature range 25 ^{o}C to 95 ^{o}C and return) in sodium cacodylate buffer, pH 7, I = 0.05 M.

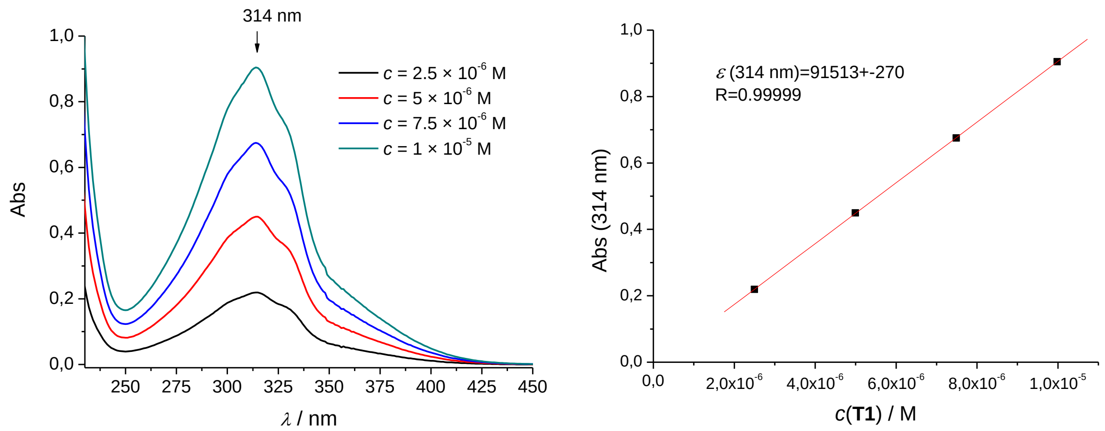

a

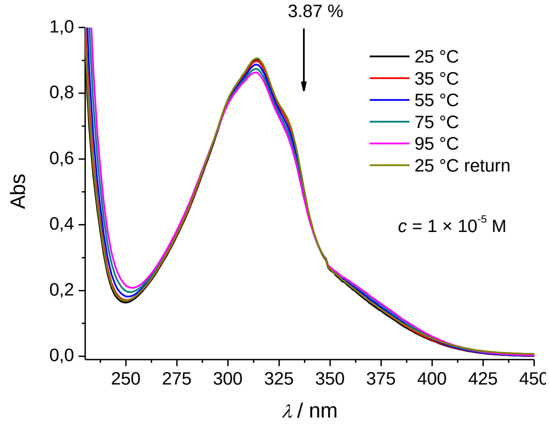

b

Figure S2. UV/Vis spectra changes of **T1**: a) Concentration dependence (concentration range 2.5 × 10^{-6} – 1 × 10^{-5} M), and b) Temperature dependence (temperature range 25 ^{o}C to 95 ^{o}C and return) in sodium cacodylate buffer, pH 7, *I* = 0.05 M.

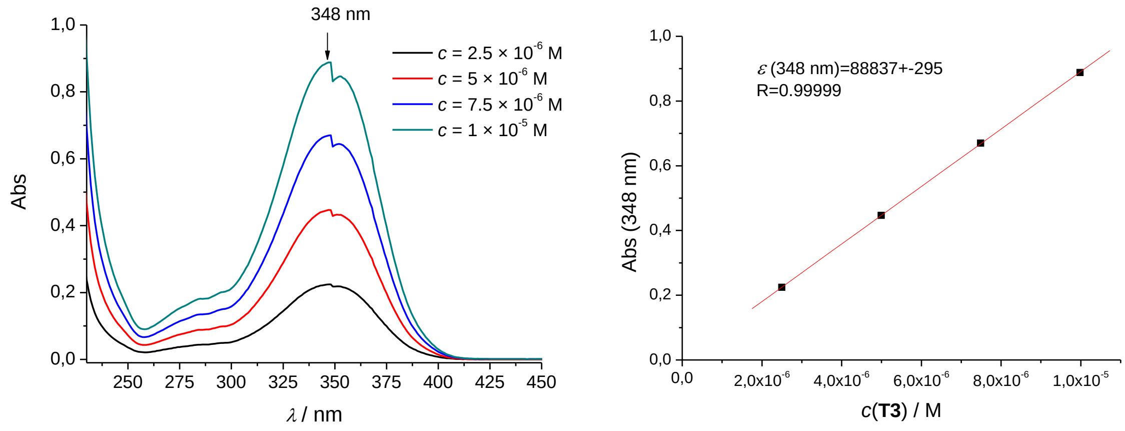

a

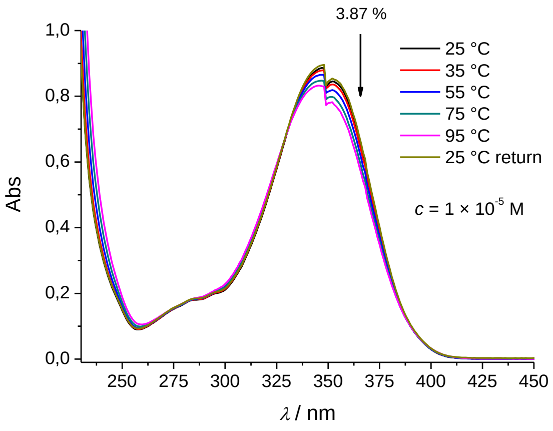

b

Figure S3. UV/Vis spectra changes of **T3**: a) Concentration dependence (concentration range $2.5 \times 10^{-6} – 1 \times 10^{-5}$ M), and b) Temperature dependence (temperature range 25 ^{o}C to 95 ^{o}C and return) in sodium cacodylate buffer, pH 7, I = 0.05 M.

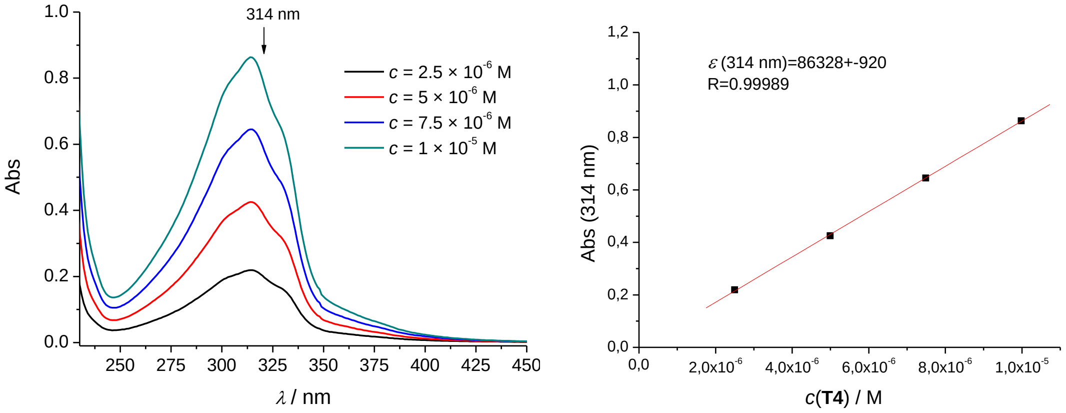

a

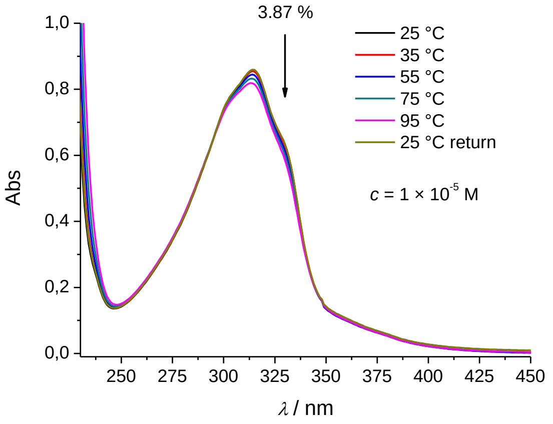

b

Figure S4. UV/Vis spectra changes of **T4**: a) Concentration dependence (concentration range $2.5 \times 10^{-6} – 1 \times 10^{-5}$ M), and b) Temperature dependence (temperature range 25 ^{o}C to 95 ^{o}C and return) in sodium cacodylate buffer, pH 7, I = 0.05 M.

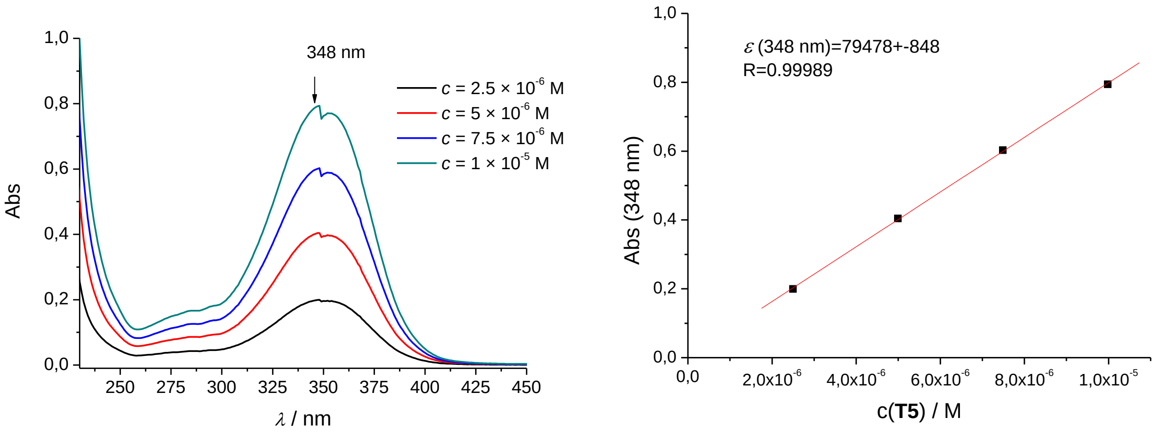

a

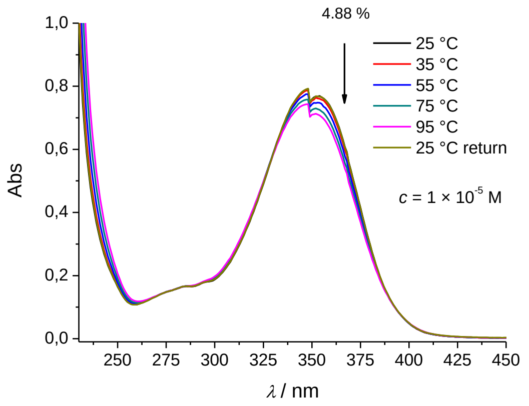

b

Figure S5. UV/Vis spectra changes of **T5**: a) Concentration dependence (concentration range 2.5 × 10^{-6} – 1 × 10^{-5} M), and b) Temperature dependence (temperature range 25 ^{o}C to 95 ^{o}C and return) in sodium cacodylate buffer, pH 7, *I* = 0.05 M.

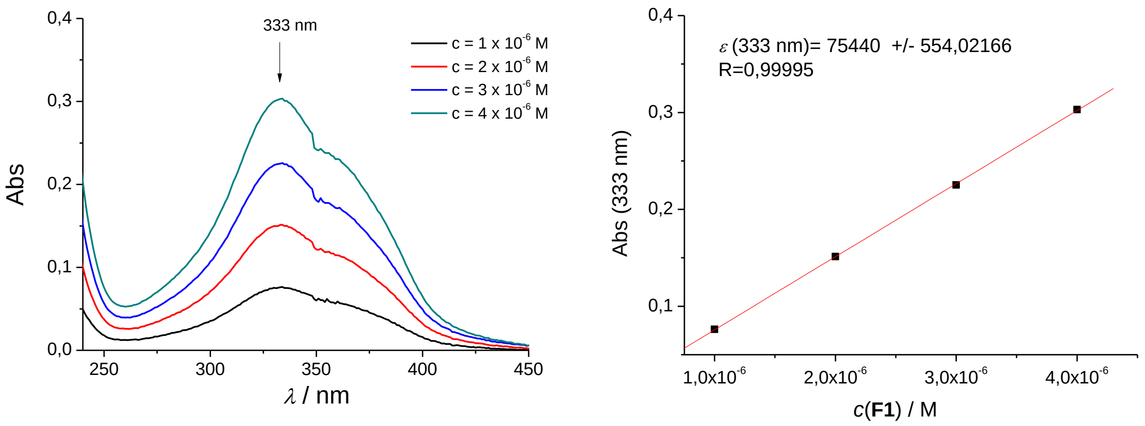

a

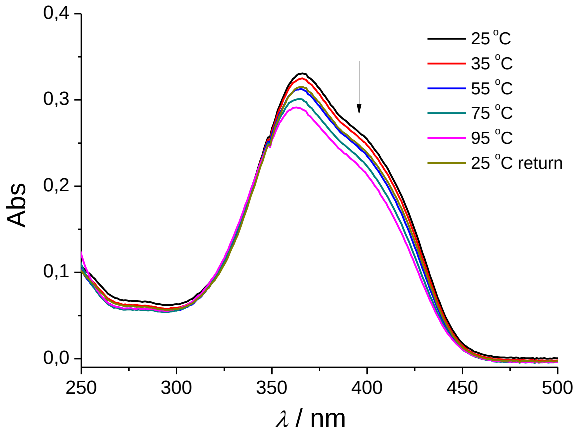

b

Figure S6. UV/Vis spectra changes of **F1**: a) Concentration dependence (concentration range 1 × 10^{-6} – 4 × 10^{-6} M), and b) Temperature dependence (temperature range 25 °C to 95 °C and back) in sodium cacodylate buffer, pH 7, *I* = 0.05 M.

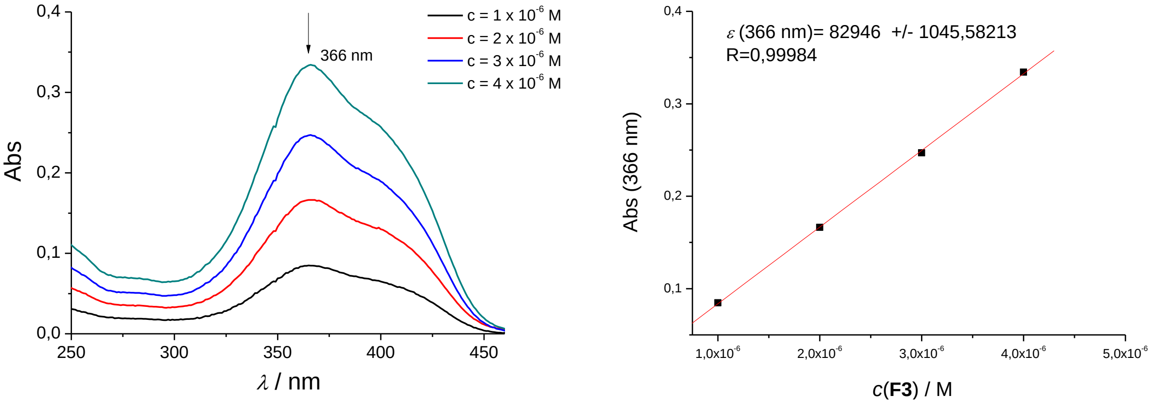

a

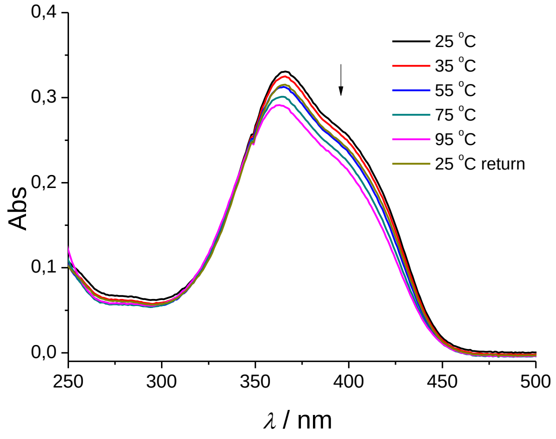

b

Figure S7. UV/Vis spectra changes of **F3**: a) Concentration dependence (concentration range 1 × 10^{-6} – 4 × 10^{-6} M), and b) Temperature dependence (temperature range 25 °C to 95 °C and back) in sodium cacodylate buffer, pH 7, *I* = 0.05 M.

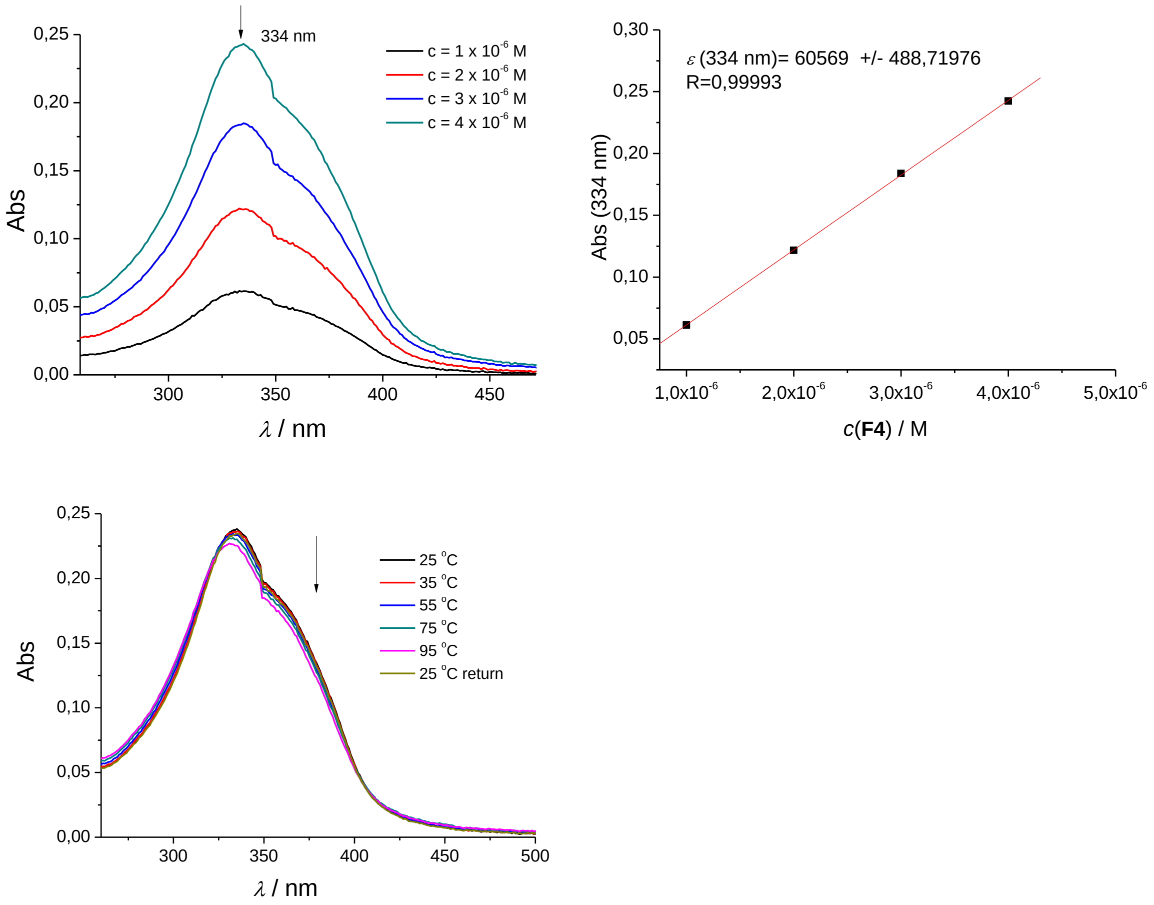

a

b

Figure S8. UV/Vis spectra changes of **F4**: a) Concentration dependence (concentration range 1 × 10^{-6} – 4 × 10^{-6} M), and b) Temperature dependence (temperature range 25 °C to 95 °C and back) in sodium cacodylate buffer, pH 7, *I* = 0.05 M.

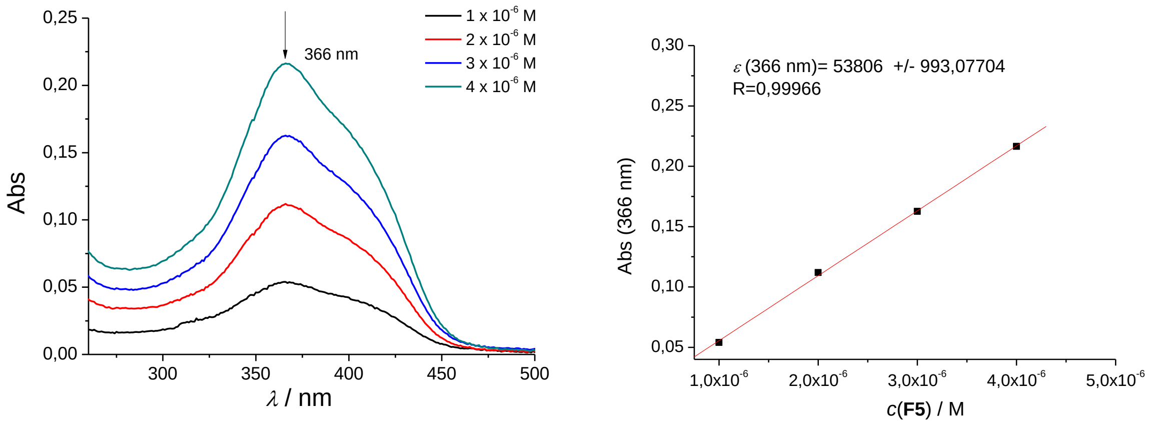

a

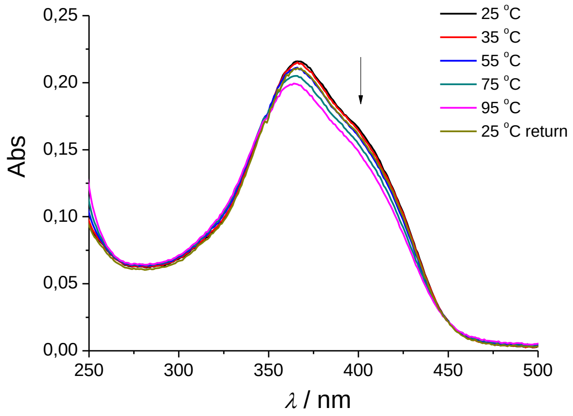

b

Figure S9. UV/Vis spectra changes of **F5**: a) Concentration dependence (concentration range 1 × 10^{-6} – 4 × 10^{-6} M), and b) Temperature dependence (temperature range 25 °C to 95 °C and back) in sodium cacodylate buffer, pH 7, *I* = 0.05 M.

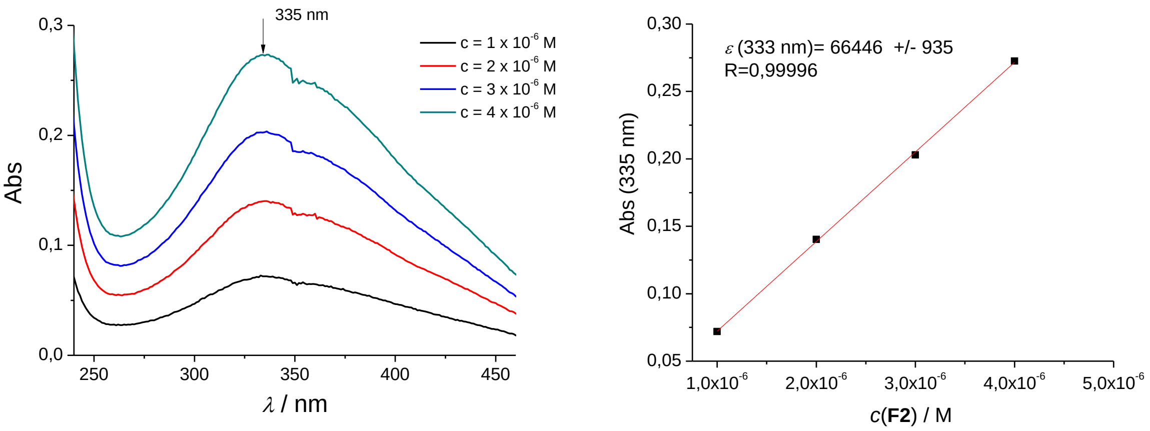

a

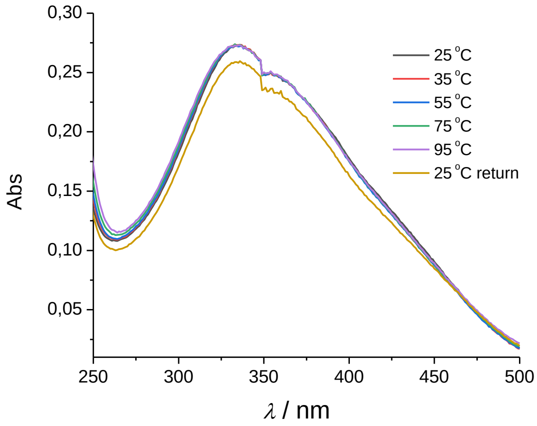

b

Figure S10. UV/Vis spectra changes of **F2**: a) Concentration dependence (concentration range 1 × 10^{-6} – 4 × 10^{-6} M), and b) Temperature dependence

(temperature range 25 ºC to 95 ºC and back) in sodium cacodylate buffer, pH 7, *I* = 0.05 M.

1.3. Fluorescence spectra

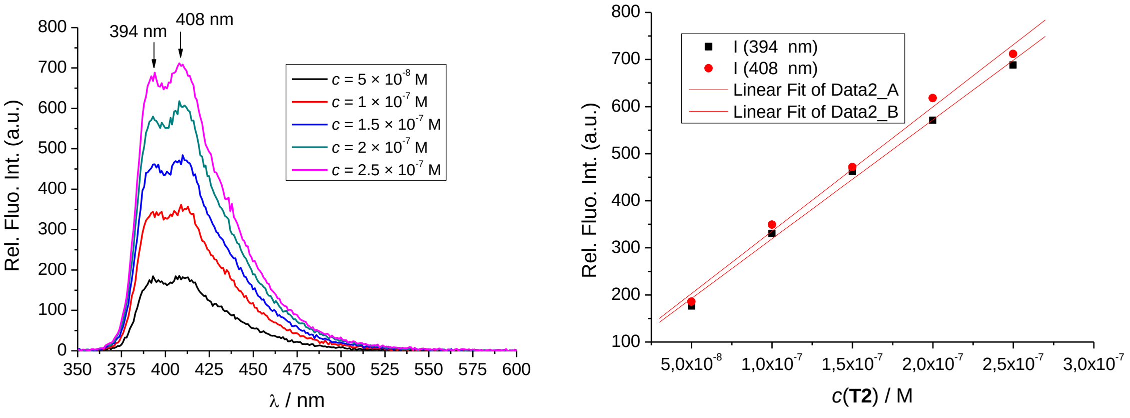

a

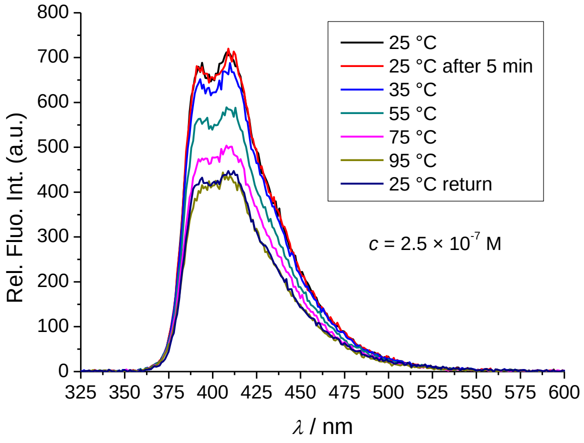

b

Figure S11. Fluorescence spectra changes of **T2**, λ_{exc} = 315 nm: a) Concentration dependence (concentration range from 5 × 10^{-8} – 2.5 × 10^{-7} M), and b) Temperature dependence (temperature range 25 ºC to 95 ºC and return) in sodium cacodylate buffer, pH 7, *I* = 0.05 M.

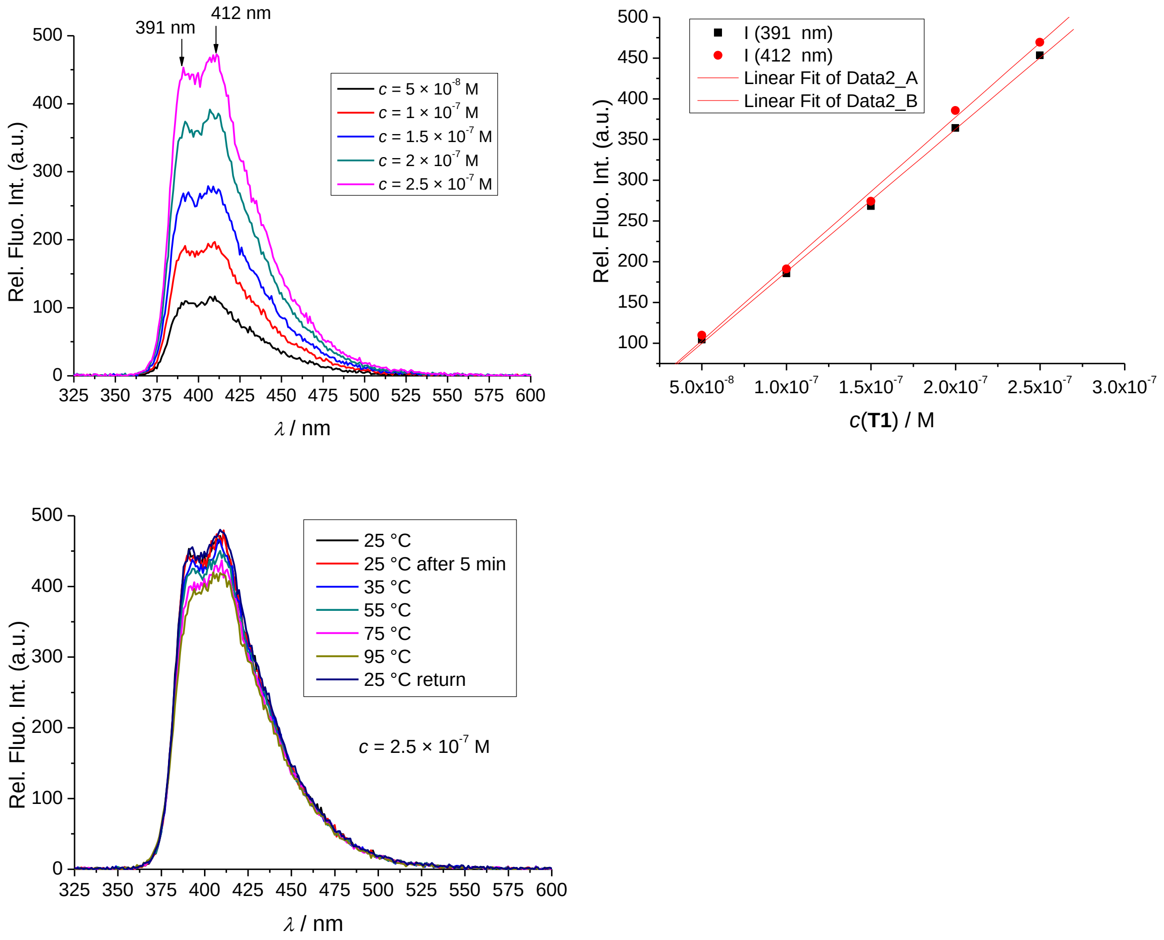

a

b

Figure S12. Fluorescence spectra changes of **T1**, λ_{exc} = 314 nm: a) Concentration dependence (concentration range from 5 × 10^{-8} – 2.5 × 10^{-7} M), and b) Temperature dependence (temperature range 25 °C to 95 °C and return) in sodium cacodylate buffer, pH 7, *I* = 0.05 M.

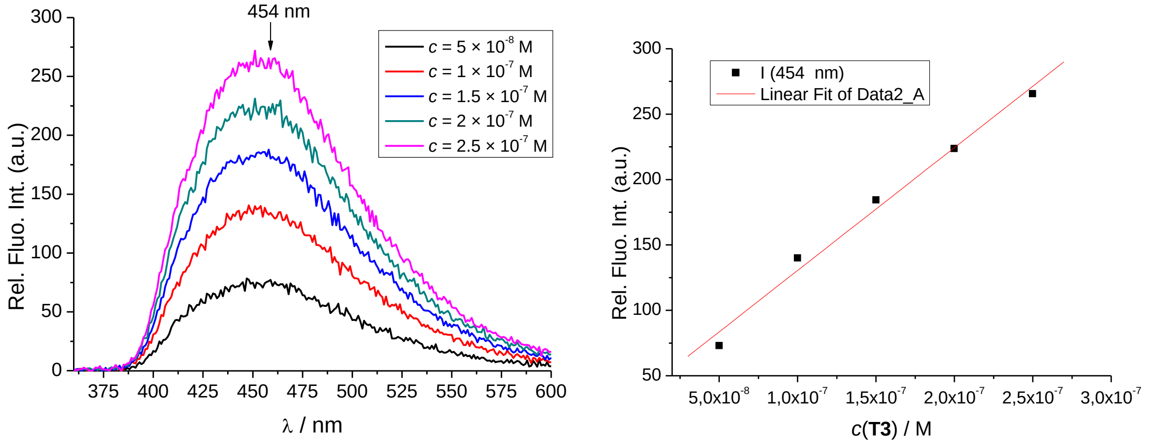

a

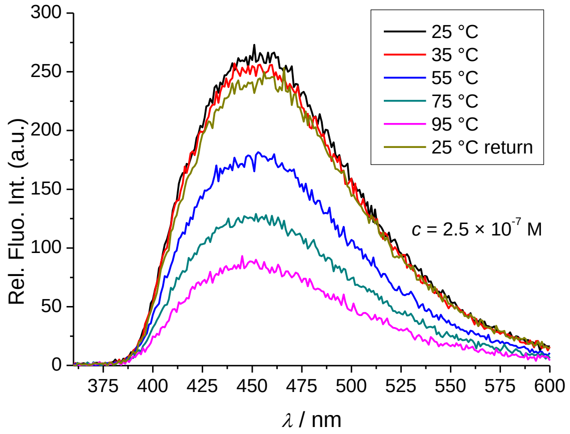

b

Figure S13. Fluorescence spectra changes of **T3**, λ_{exc} = 348 nm: a) Concentration dependence (concentration range from 5 × 10^{-8} – 2.5 × 10^{-7} M), and b) Temperature dependence (temperature range 25 °C to 95 °C and return) in sodium cacodylate buffer, pH 7, I = 0.05 M.

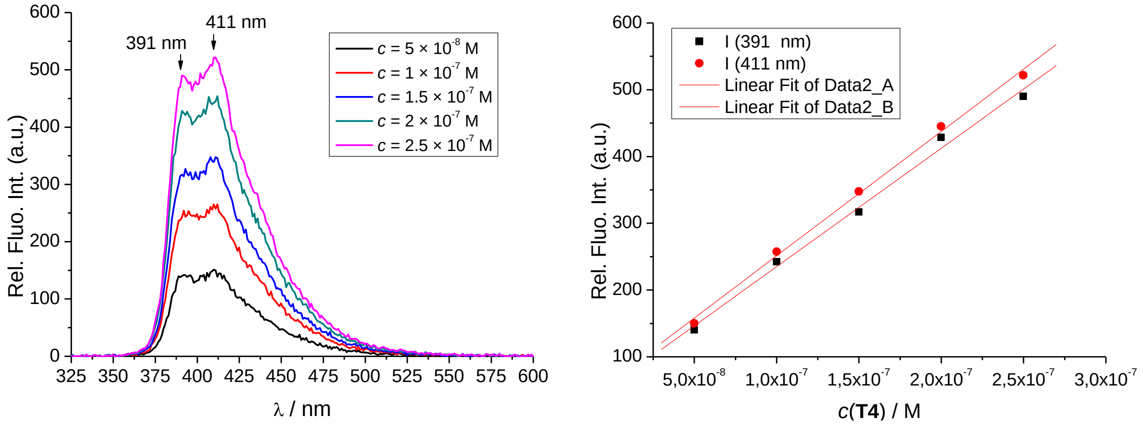

a

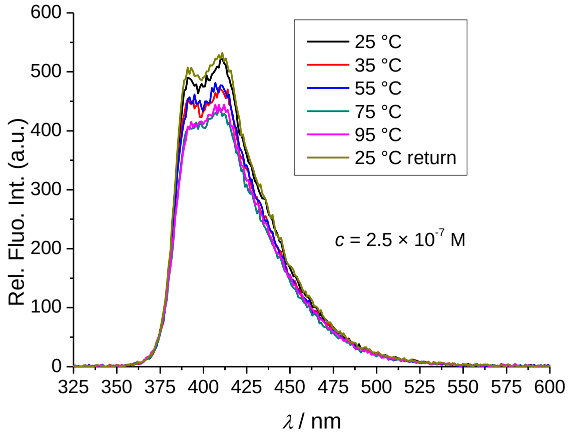

b

Figure S14. Fluorescence spectra changes of **T4**, λ_{exc} = 314 nm: a) Concentration dependence (concentration range from 5 × 10^{-8} – 2.5 × 10^{-7} M), and b) Temperature

dependence (temperature range 25 °C to 95 °C and return) in sodium cacodylate buffer, pH 7, I = 0.05 M.

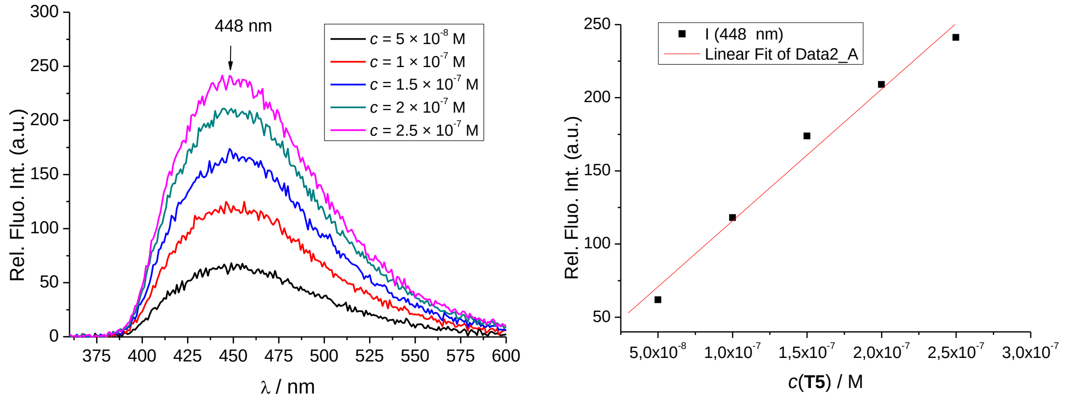

a

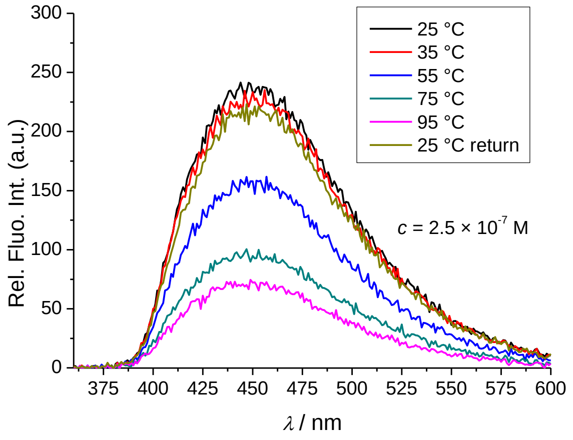

b

Figure S15. Fluorescence spectra changes of **T5**, λ_{exc} = 348 nm: a) Concentration dependence (concentration range from 5 × 10^{-8} – 2.5 × 10^{-7} M), and b) Temperature dependence (temperature range 25 °C to 95 °C and return) in sodium cacodylate buffer, pH 7, I = 0.05 M.

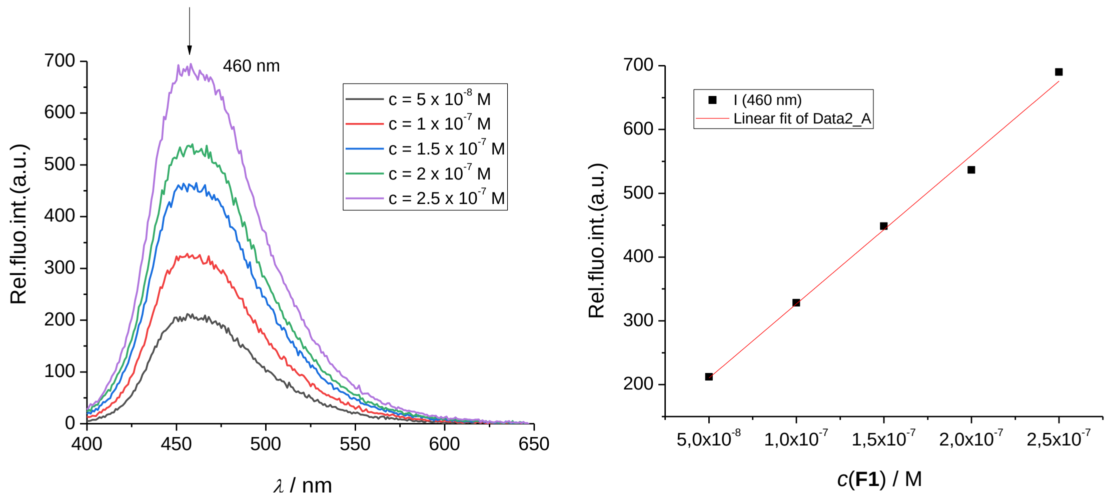

a

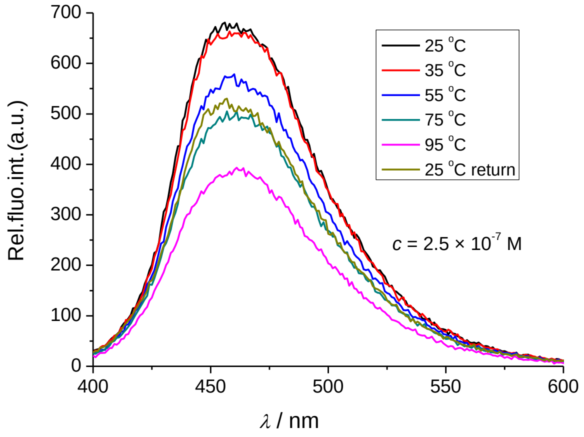

b

Figure S16. Fluorescence spectra changes of **F1**, λ_{exc} = 333 nm: a) Concentration dependence (concentration range from 5 × 10^{-8} – 2.5 × 10^{-7} M), and b) Temperature dependence (temperature range 25 ºC to 95 ºC and back) in sodium cacodylate buffer, pH 7, *I* = 0.05 M.

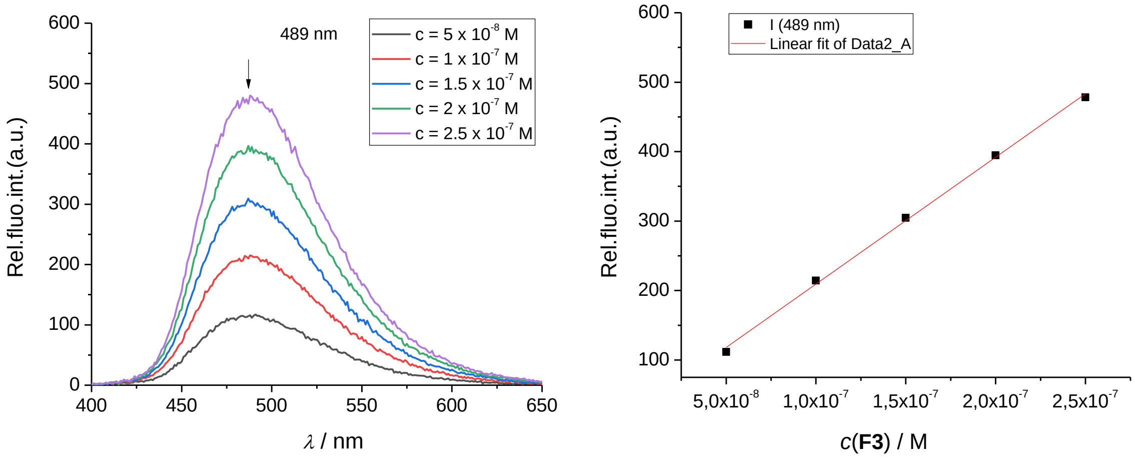

a

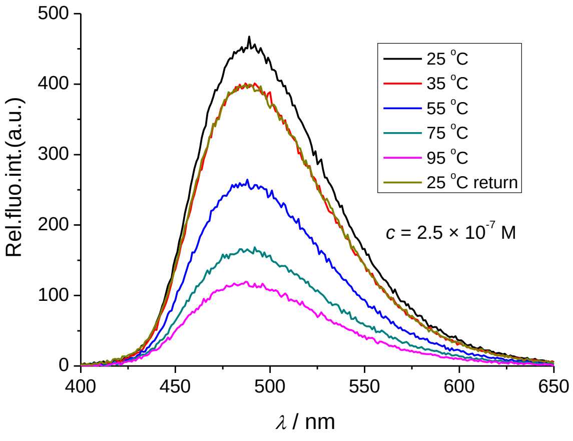

b

Figure S17. Fluorescence spectra changes of **F3**, λ_{exc} = 366 nm: a) Concentration dependence (concentration range from 5 × 10^{-8} – 2.5 × 10^{-7} M), and b) Temperature

dependence (temperature range 25 ºC to 95 ºC and back) in sodium cacodylate buffer, pH 7, *I* = 0.05 M.

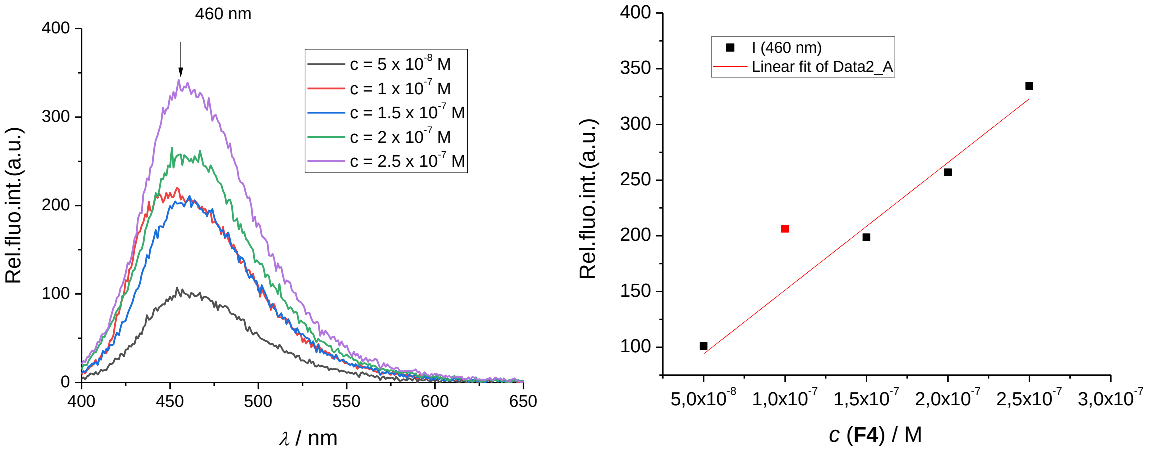

a

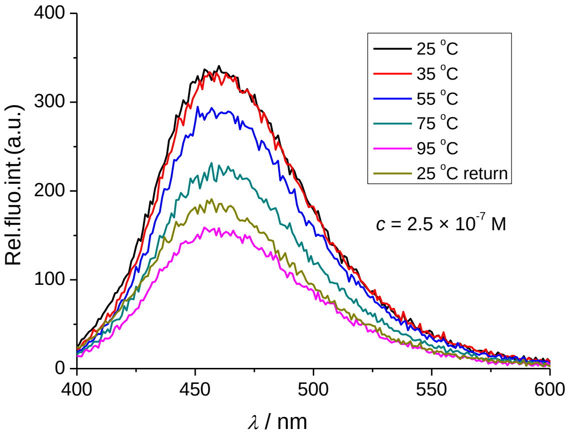

b

Figure S18. Fluorescence spectra changes of **F4**, λ_{exc} = 334 nm: a) Concentration dependence (concentration range from 5 × 10^{-8} – 2.5 × 10^{-7} M), and b) Temperature dependence (temperature range 25 ºC to 95 ºC and back) in sodium cacodylate buffer, pH 7, *I* = 0.05 M.

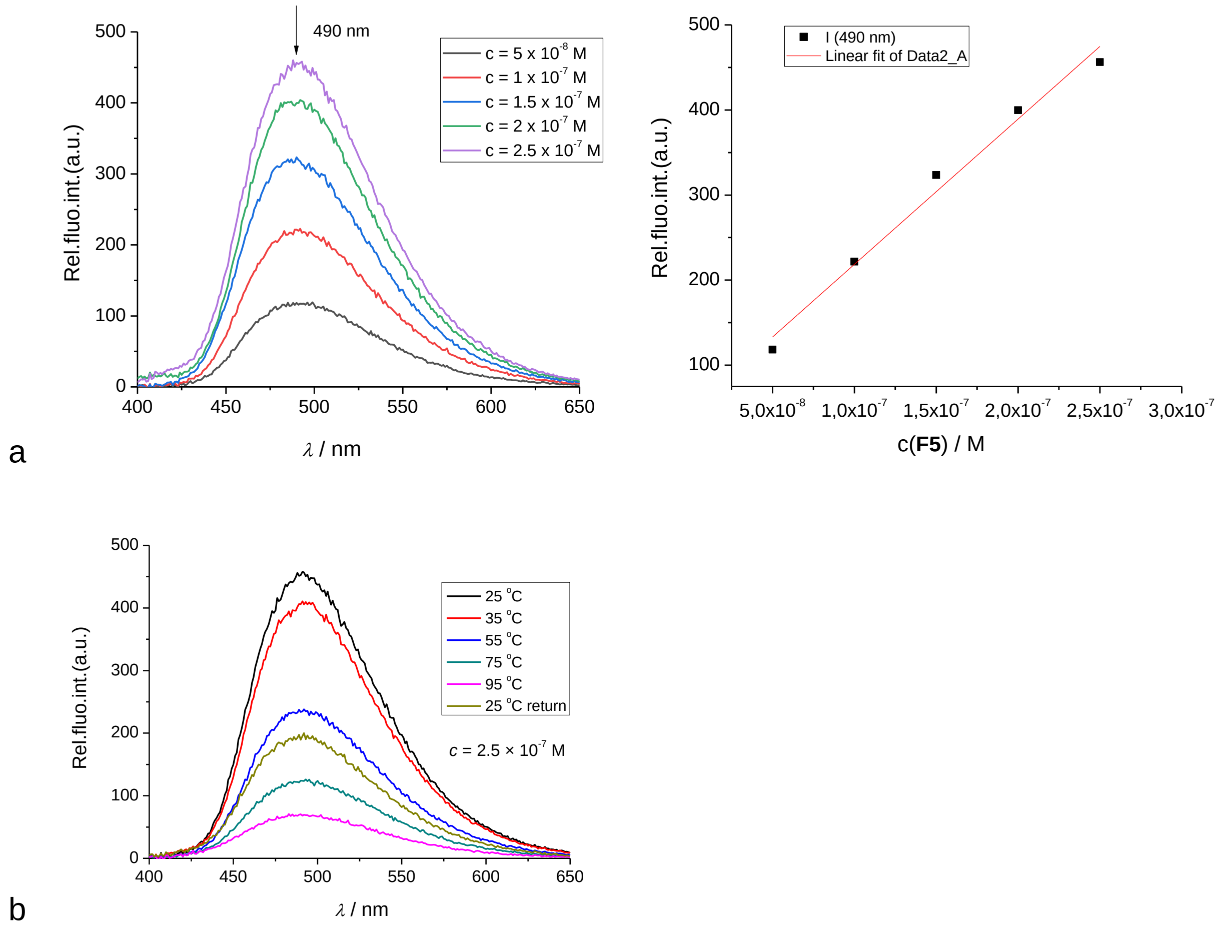

Figure S19. Fluorescence spectra changes of **F5**, λ_{exc} = 366 nm: a) Concentration dependence (concentration range from 5 × 10^{-8} – 2.5 × 10^{-7} M), and b) Temperature dependence (temperature range 25 °C to 95 °C and back) in sodium cacodylate buffer, pH 7, I = 0.05 M.

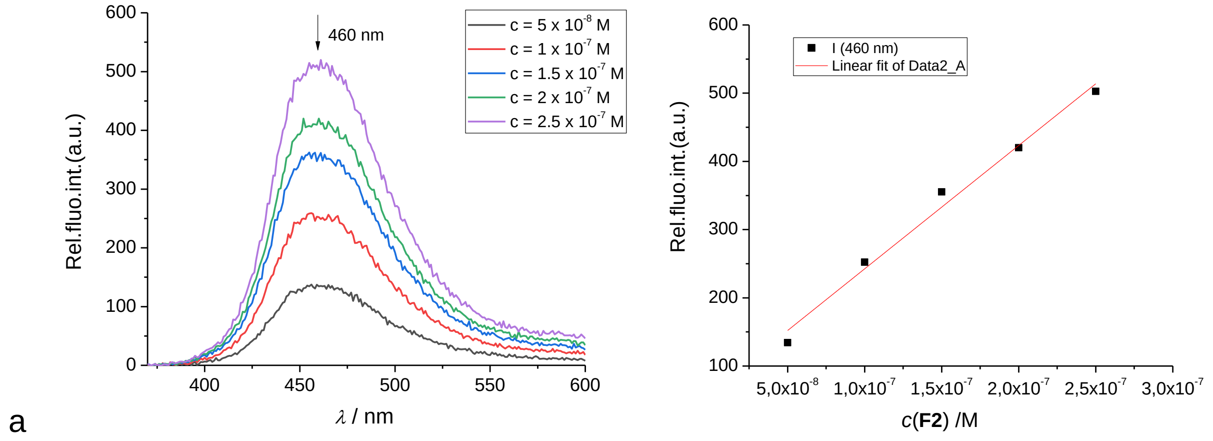

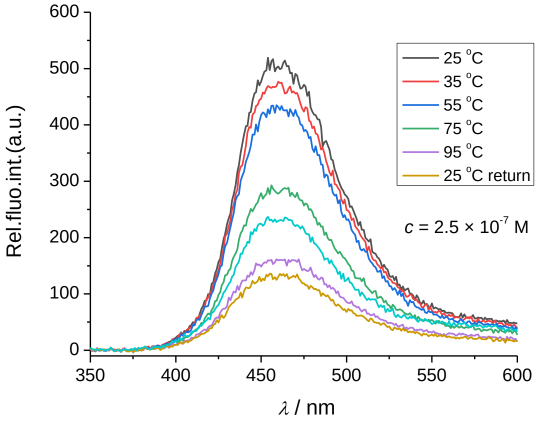

b

Figure S20. Fluorescence spectra changes of **F2**, λ_{exc} = 335 nm: a) Concentration dependence (concentration range from 5 × 10^{-8} – 2.5 × 10^{-7} M), and b) Temperature dependence (temperature range 25 °C to 95 °C and back) in sodium cacodylate buffer, pH 7, I = 0.05 M.

2. Study of interactions of T1-T5 and F1-F5 with DNA/RNA (ctDNA and pApU) in buffered solution (sodium cacodylate pH = 7)

Table S1. Structural properties of studied ds-DNA and ds-RNA [1,2].

Structure type	Groove width [Å]		Groove depth [Å]	
	major	minor	major	minor
[a] poly rA – poly rU	3.8	10.9	13.5	2.8
[b] ct-DNA (48% of GC-pairs)	11.4	3.3	7.5	7.9
[b] poly dAdT – poly dAdT	11.2	6.3	8.5	7.5
[c] poly dGdC – poly dGdC	13.5	9.5	10.0	7.2

[a] A - helical structure

[b] B - helical structure

[c] B - helical structure with sterically blocked minor groove by amino groups of guanines

2.1. Thermal denaturation experiments

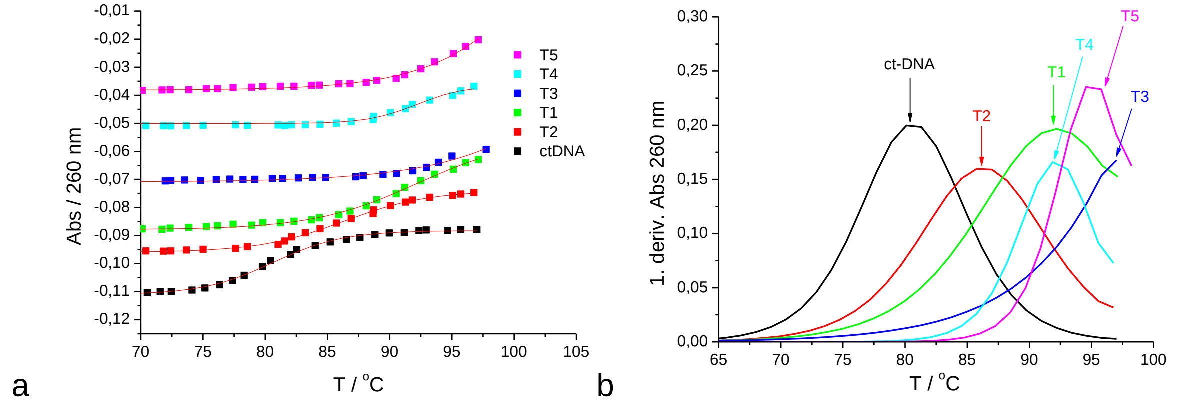

Figure S21. a) Denaturation of **ctDNA** upon addition of *r* = 0.1 ([compound/ [polynucleotide]) of **T1-T5** at pH 7.0 (buffer sodium cacodylate, *I* = 0.05 M), red lines denote fitting of experimental data to sigmoidal eq. by Origin 7.0; b) The first derivation of absorbance (fitted to sigmoidal eq.) on temperature dependence.

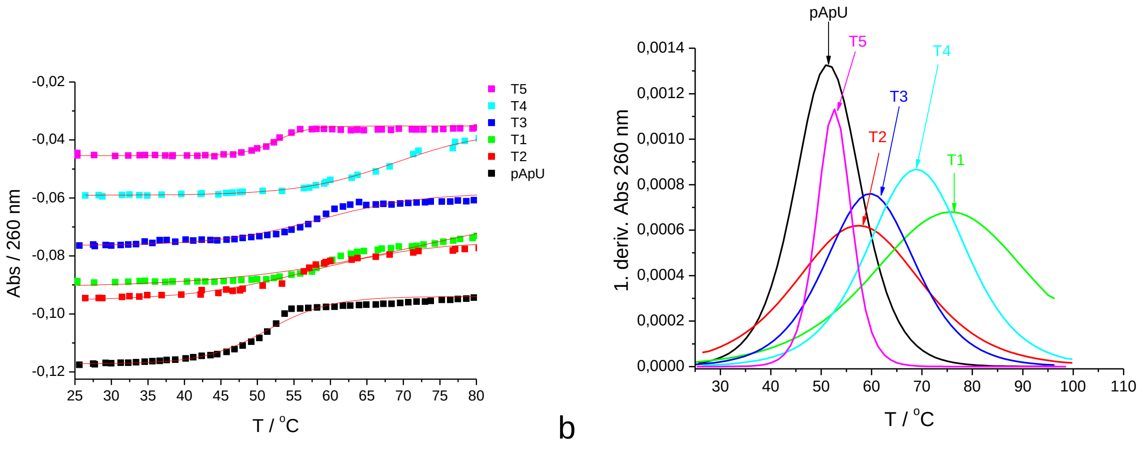

Figure S22. a) Denaturation of **poly A – poly U** upon addition of *r* = 0.1 ([compound/ [polynucleotide]) of **T1-T5** at pH 7.0 (buffer sodium cacodylate, *I* = 0.05 M), red lines denote fitting of experimental data to sigmoidal eq. by Origin 7.0; b) The first derivation of absorbance (fitted to sigmoidal eq.) on temperature dependence.

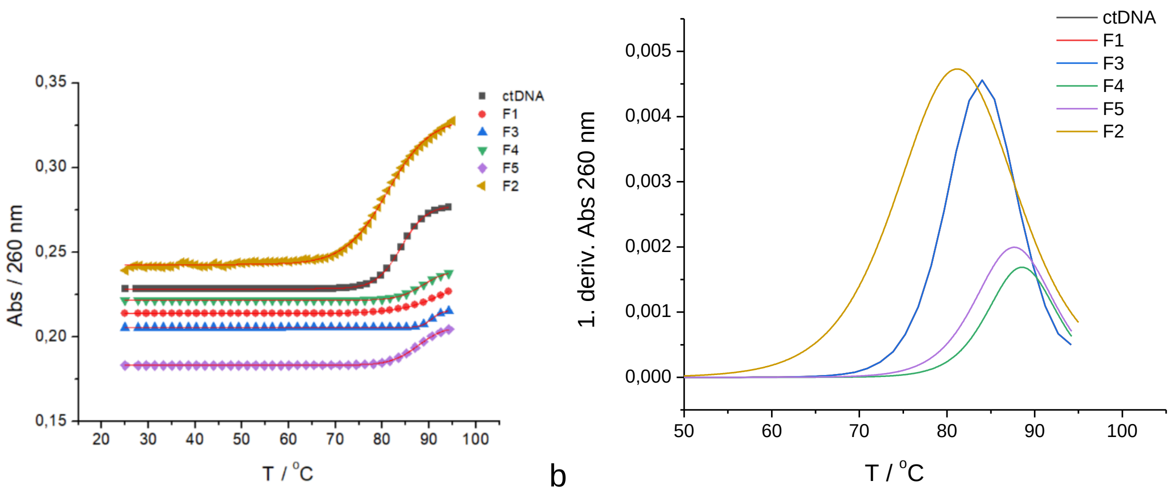

Figure S23. a) Denaturation of **ctDNA** upon addition of *r* = 0.1 ([compound/ [polynucleotide]) of **F1-F5** at pH 7.0 (buffer sodium cacodylate, *I* = 0.05 M), red lines denote fitting of experimental data to sigmoidal eq. by Origin 7.0; b) The first derivation of absorbance (fitted to sigmoidal eq.) on temperature dependence.

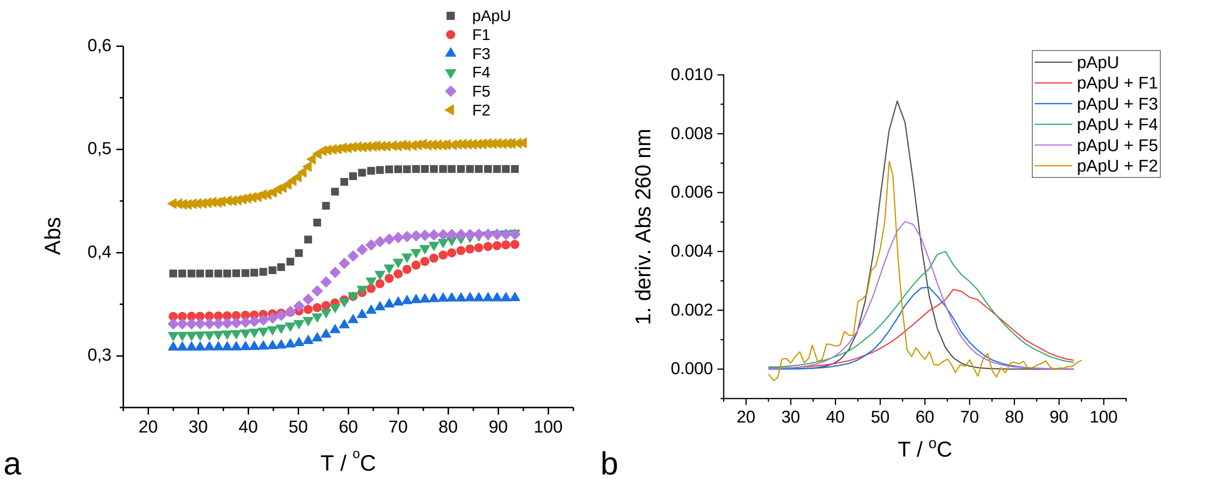

Figure S24. a) Denaturation of **poly A – poly U** upon addition of r = 0.1 ([compound]/[polynucleotide]) of **F1-F5** at pH 7.0 (buffer sodium cacodylate, I = 0.05 M), red lines denote fitting of experimental data to sigmoidal eq. by Origin 7.0; b) The first derivation of absorbance (fitted to sigmoidal eq.) on temperature dependence.

2.2. Fluorescence titration experiments

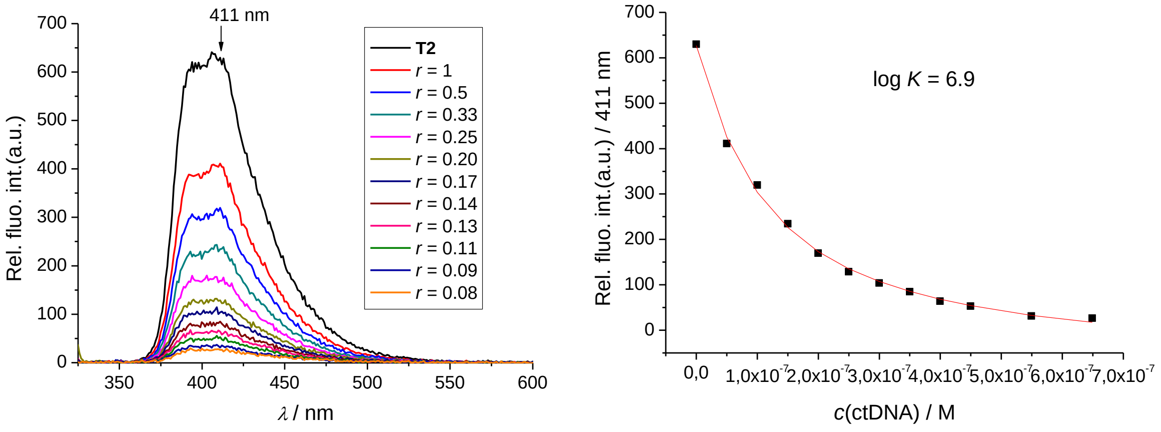

Figure S25. a) Changes in fluorescence spectrum of **T2** ($c = 5 \times 10^{-8}$ M, λ_{exc}= 315 nm) upon titration with **ctDNA** ($c = 5 \times 10^{-8} – 6.5 \times 10^{-7}$ M), slit: 5-10; b) Dependence of **T2** emission at λ_{max} = 411 nm on *c*(ctDNA), at pH 7.0, sodium cacodylate buffer, *I* = 0.05 M.

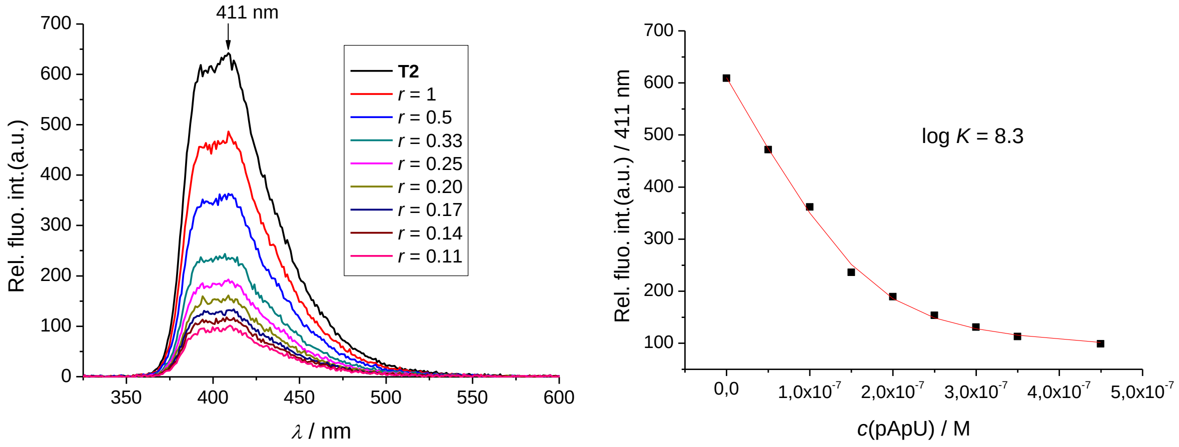

Figure S26. a) Changes in fluorescence spectrum of **T2** ($c = 5 \times 10^{-8}$ M, λ_{exc}= 315 nm) upon titration with **pApU** ($c = 5 \times 10^{-8} – 4.5 \times 10^{-7}$ M), slit: 5-10; b) Dependence of **T2** emission at λ_{max} = 411 nm on *c*(pApU), at pH 7.0, sodium cacodylate buffer, *I* = 0.05 M.

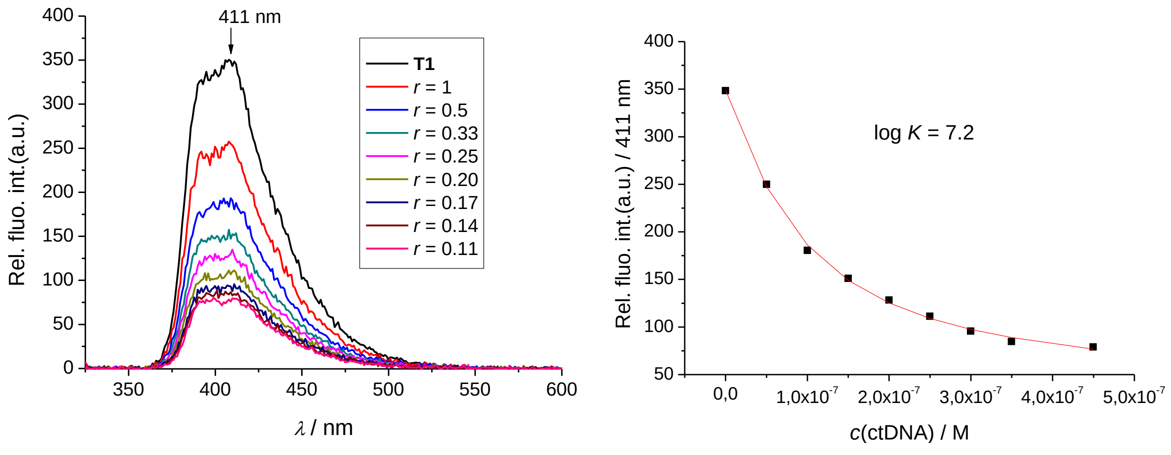

Figure S27. a) Changes in fluorescence spectrum of **T1** ($c = 5 \times 10^{-8}$ M, λ_{exc}= 314 nm) upon titration with **ctDNA** ($c = 5 \times 10^{-8} – 4.5 \times 10^{-7}$ M), slit: 5-10; b) Dependence of **T1** emission at λ_{max} = 411 nm on *c*(ctDNA), at pH 7.0, sodium cacodylate buffer, *I* = 0.05 M.

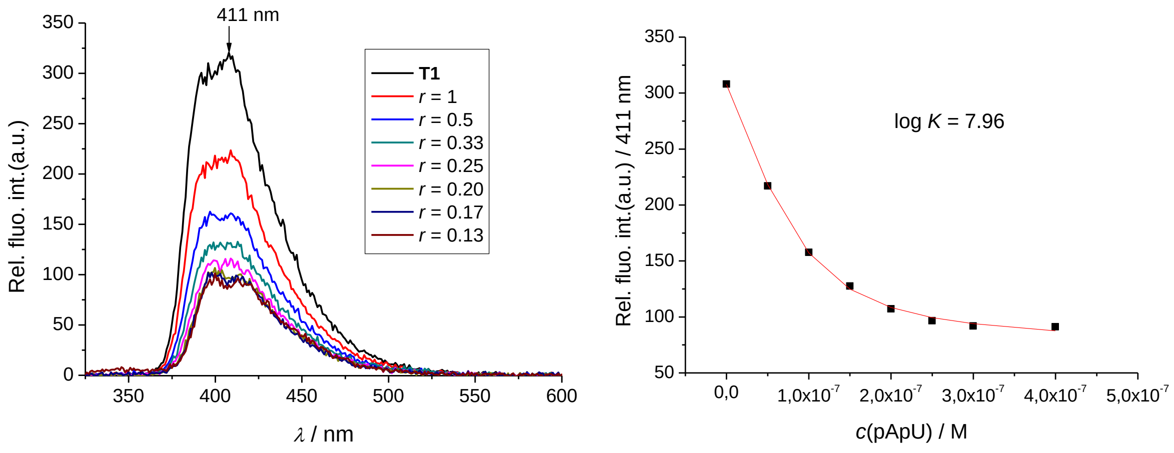

Figure S28. a) Changes in fluorescence spectrum of **T1** ($c = 5 \times 10^{-8}$ M, λ_{exc}= 314 nm) upon titration with **pApU** ($c = 5 \times 10^{-8} – 4 \times 10^{-7}$ M), slit: 5-10; b) Dependence of **T1** emission at λ_{max} = 411 nm on *c*(pApU), at pH 7.0, sodium cacodylate buffer, *I* = 0.05 M.

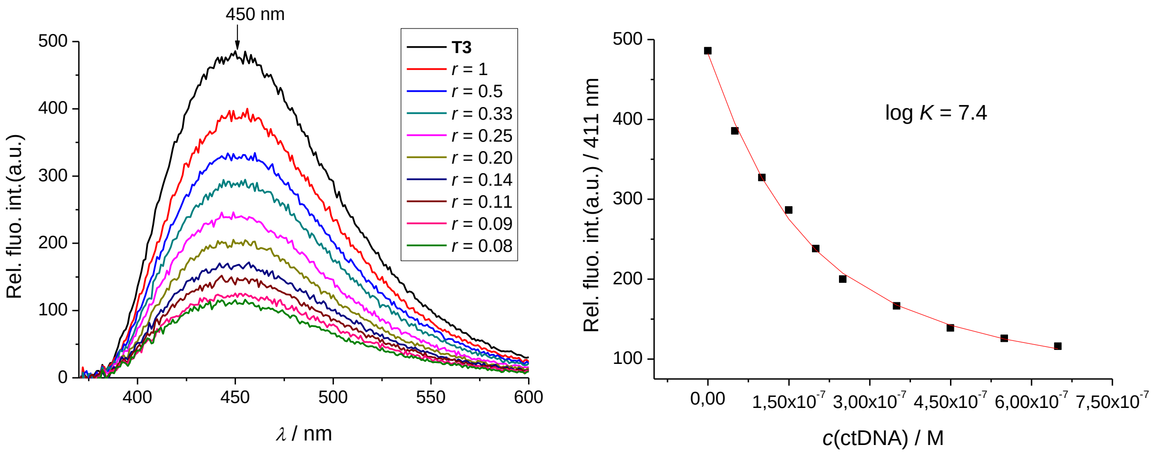

Figure S29. a) Changes in fluorescence spectrum of **T3** (c = 5 × 10^{-8} M, λ_{exc}= 348 nm) upon titration with **ctDNA** (c = 5 × 10^{-8} – 6.5 × 10^{-7} M), slit: 20-20; b) Dependence of **T3** emission at λ_{max} = 450 nm on c(ctDNA), at pH 7.0, sodium cacodylate buffer, I = 0.05 M.

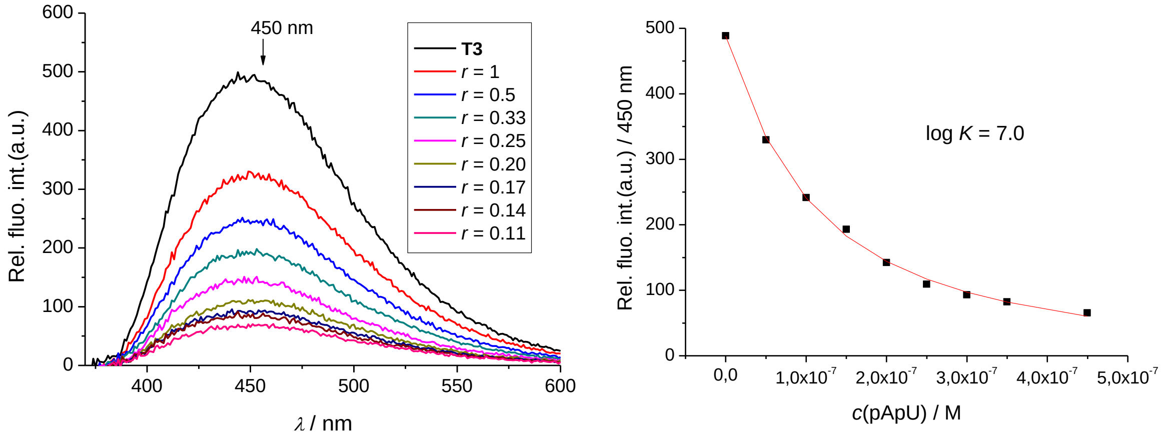

Figure S30. a) Changes in fluorescence spectrum of **T3** (c = 5 × 10^{-8} M, λ_{exc}= 348 nm) upon titration with **pApU** (c = 5 × 10^{-8} – 4.5 × 10^{-7} M), slit: 20-20; b) Dependence of **T3** emission at λ_{max} = 450 nm on c(pApU), at pH 7.0, sodium cacodylate buffer, I = 0.05 M.

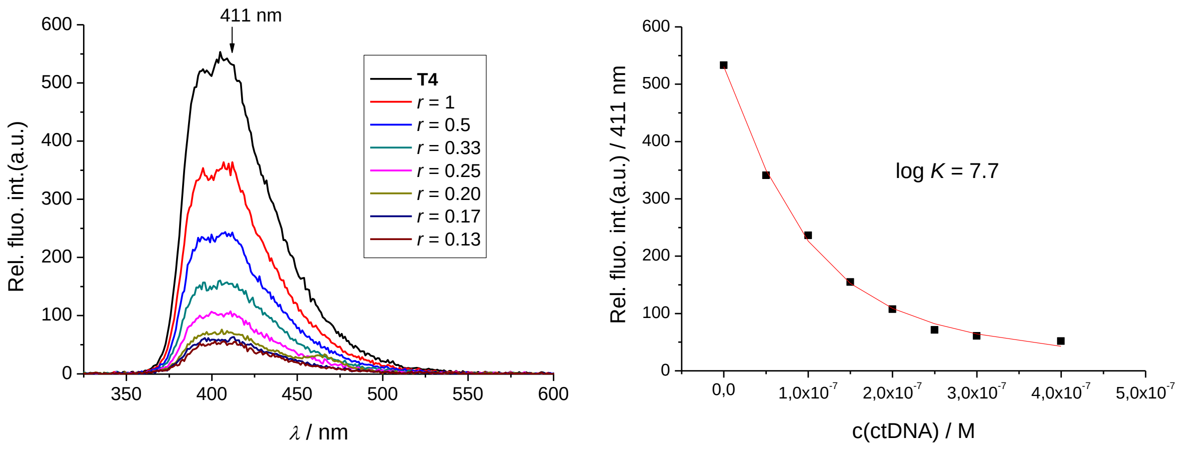

Figure S31. a) Changes in fluorescence spectrum of **T4** ($c = 5 \times 10^{-8}$ M, λ_{exc}= 314 nm) upon titration with **ctDNA** ($c = 5 \times 10^{-8} – 4 \times 10^{-7}$ M), slit: 5-10; b) Dependence of **T4** emission at λ_{max} = 411 nm on *c*(ctDNA), at pH 7.0, sodium cacodylate buffer, *I* = 0.05 M.

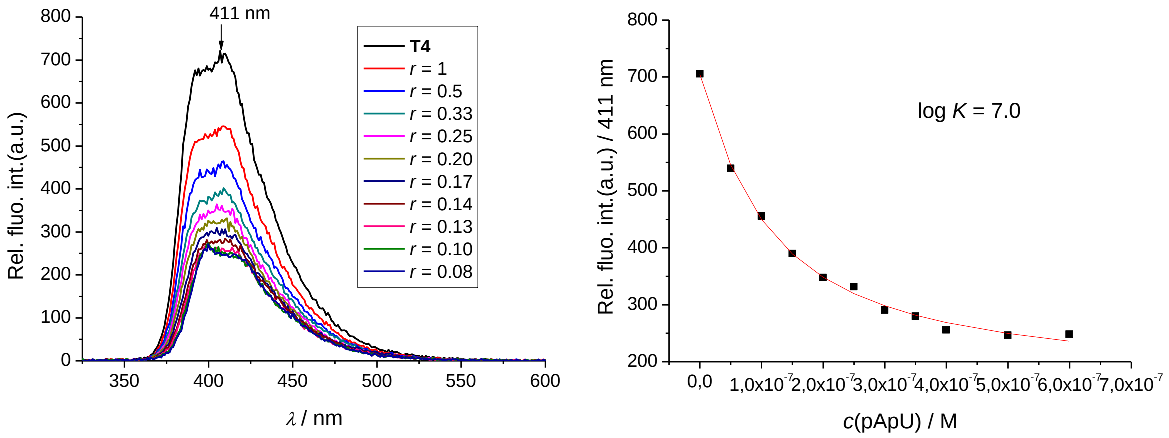

Figure S32. a) Changes in fluorescence spectrum of **T4** ($c = 2.5 \times 10^{-8}$ M, λ_{exc}= 314 nm) upon titration with **pApU** ($c = 5 \times 10^{-8} – 6 \times 10^{-7}$ M), slit: 5-10; b) Dependence of **T4** emission at λ_{max} = 411 nm on *c*(pApU), at pH 7.0, sodium cacodylate buffer, *I* = 0.05 M.

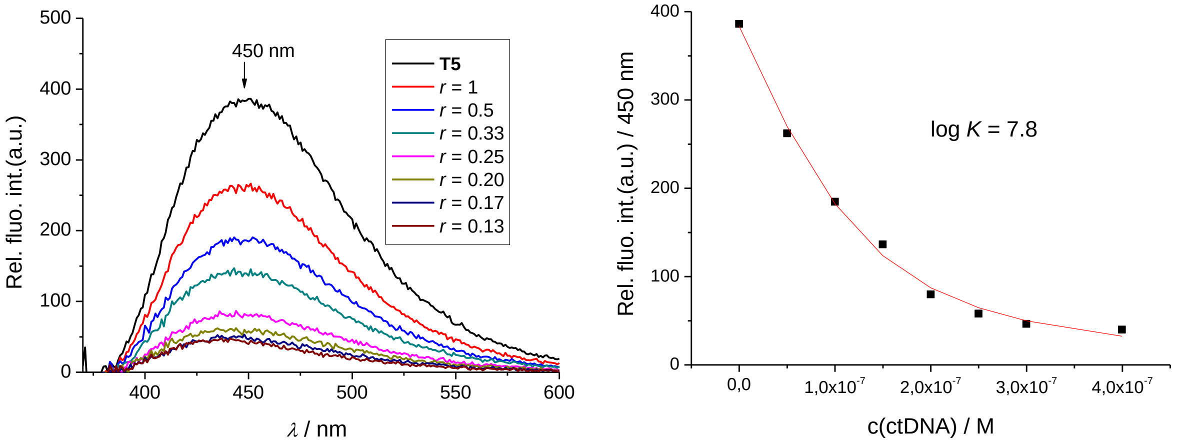

Figure S33. a) Changes in fluorescence spectrum of **T5** (c = 5 × 10^{-8} M, λ_{exc}= 348 nm) upon titration with **ctDNA** (c = 5 × 10^{-8} – 4 × 10^{-7} M), slit: 20-20; b) Dependence of **T5** emission at λ_{max} = 450 nm on c(ctDNA), at pH 7.0, sodium cacodylate buffer, I = 0.05 M.

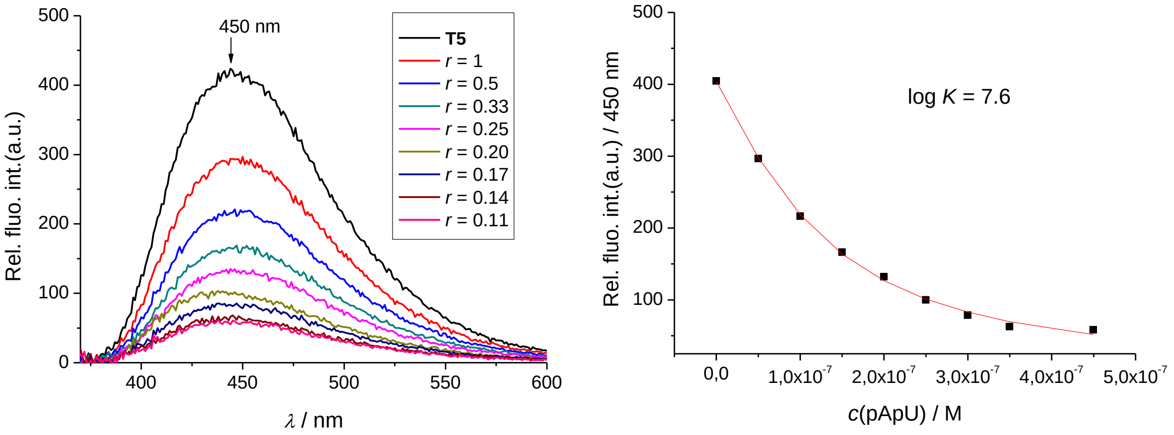

Figure S34. a) Changes in fluorescence spectrum of **T5** (c = 5 × 10^{-8} M, λ_{exc}= 348 nm) upon titration with **pApU** (c = 5 × 10^{-8} – 4.5 × 10^{-7} M), slit: 20-20 b) Dependence of **T5** emission at λ_{max} = 450 nm on c(pApU), at pH 7.0, sodium cacodylate buffer, I = 0.05 M.

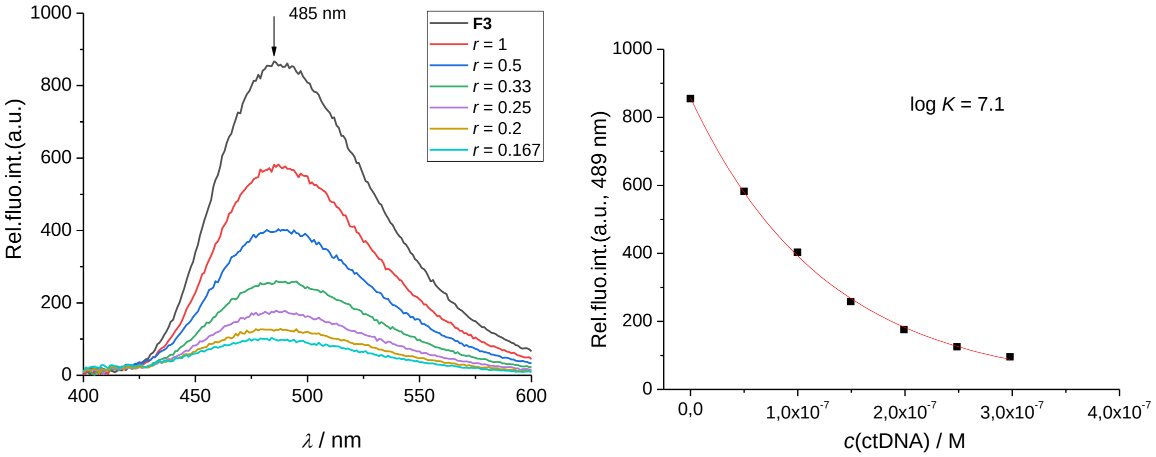

Figure S35. a) Changes in fluorescence spectrum of **F3** (c = 5 × 10^{-8} M, λ_{exc}= 366 nm) upon titration with **ctDNA** (c = 5 × 10^{-8} – 3 × 10^{-7} M), slit: 20-20; b) Dependence of **F3** emission at λ_{max} = 485 nm on c(ctDNA), at pH 7.0, sodium cacodylate buffer, I = 0.05 M.

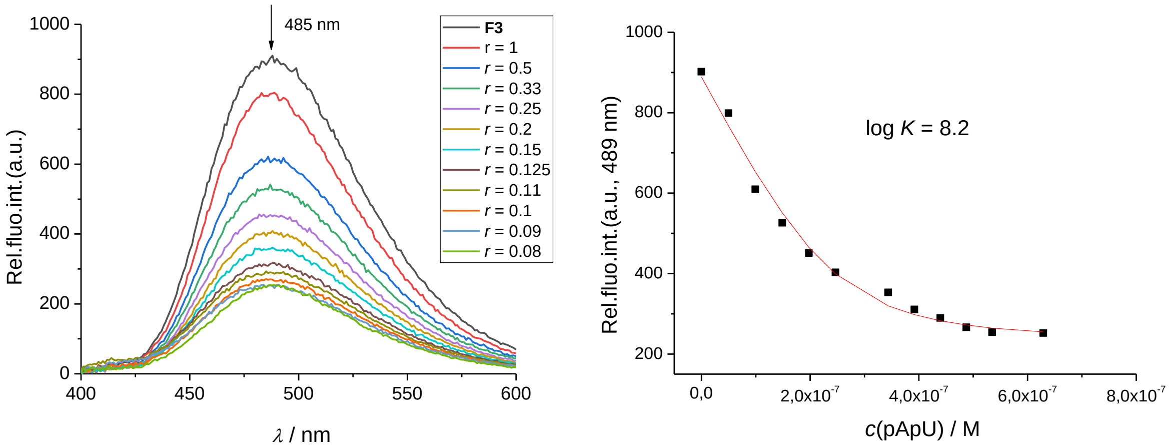

Figure S36. a) Changes in fluorescence spectrum of **F3** (c = 5 × 10^{-8} M, λ_{exc}= 366 nm) upon titration with **pApU** (c = 5 × 10^{-8} – 6.3 × 10^{-7} M), slit: 20-20; b) Dependence of **F3** emission at λ_{max} = 485 nm on c(pApU), at pH 7.0, sodium cacodylate buffer, I = 0.05 M.

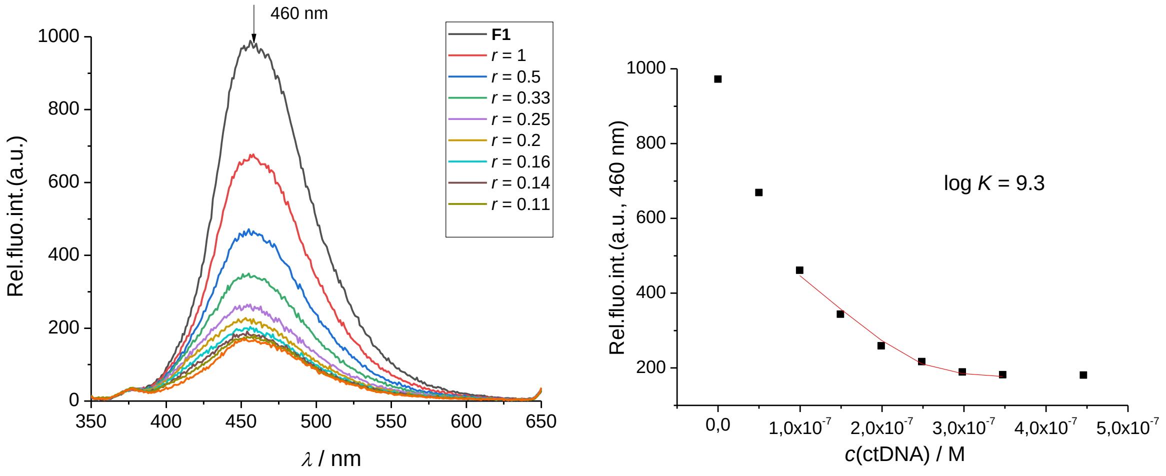

Figure S37. a) Changes in fluorescence spectrum of **F1** ($c = 5 \times 10^{-8}$ M, λ_{exc}= 333 nm) upon titration with **ctDNA** ($c = 5 \times 10^{-8} – 4.5 \times 10^{-7}$ M), slit: 10-10; b) Dependence of **F1** emission at λ_{max} = 460 nm on *c*(ctDNA), at pH 7.0, sodium cacodylate buffer, *I* = 0.05 M.

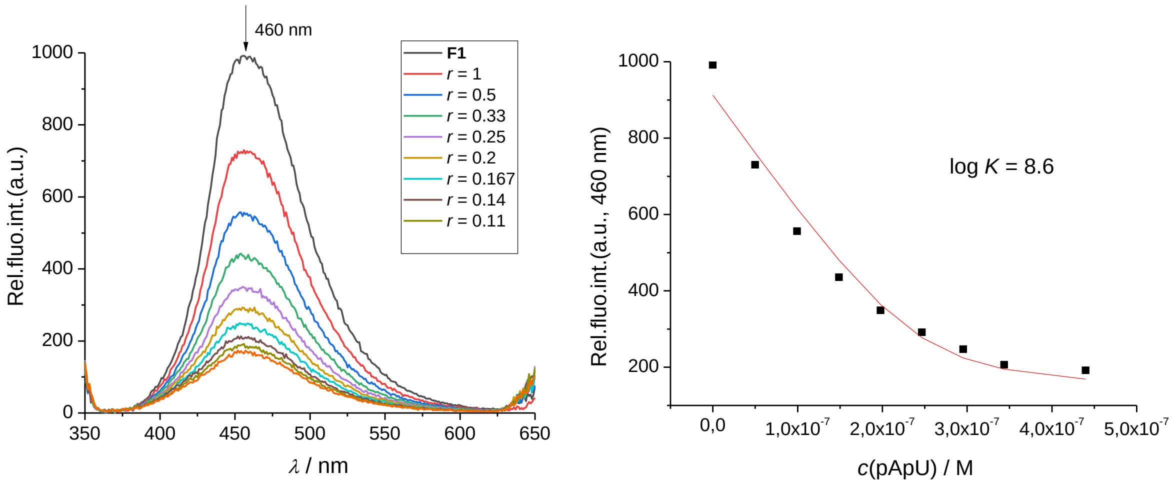

Figure S38. a) Changes in fluorescence spectrum of **F1** ($c = 5 \times 10^{-8}$ M, λ_{exc}= 333 nm) upon titration with **pApU** ($c = 5 \times 10^{-8} – 4.4 \times 10^{-7}$ M), slit: 20-10; b) Dependence of **F1** emission at λ_{max} = 460 nm on *c*(pApU), at pH 7.0, sodium cacodylate buffer, *I* = 0.05 M.

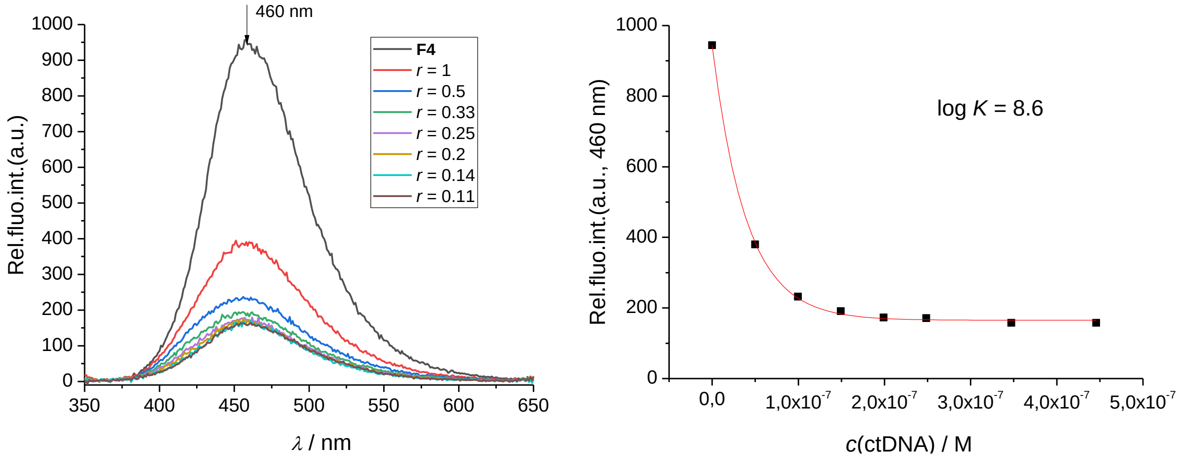

Figure S39. a) Changes in fluorescence spectrum of **F4** ($c = 5 \times 10^{-8}$ M, λ_{exc}= 334 nm) upon titration with **ctDNA** ($c = 5 \times 10^{-8} – 4.5 \times 10^{-7}$ M), slit: 10-20; b) Dependence of **F4** emission at λ_{max} = 460 nm on c(ctDNA), at pH 7.0, sodium cacodylate buffer, I = 0.05 M.

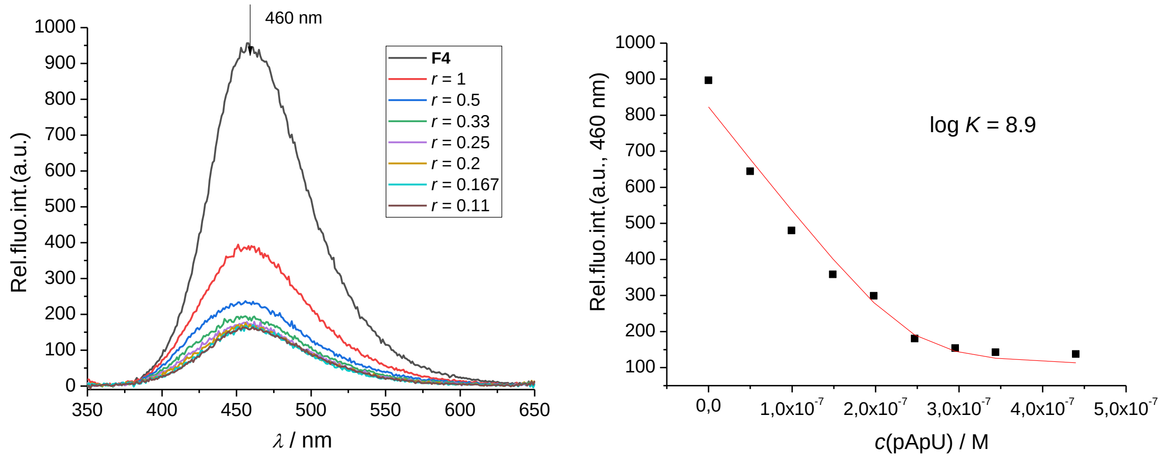

Figure S40. a) Changes in fluorescence spectrum of **F4** ($c = 5 \times 10^{-8}$ M, λ_{exc}= 334 nm) upon titration with **pApU** ($c = 5 \times 10^{-8} – 4.5 \times 10^{-7}$ M), slit: 10-20; b) Dependence of **F4** emission at λ_{max} = 460 nm on c(pApU), at pH 7.0, sodium cacodylate buffer, I = 0.05 M.

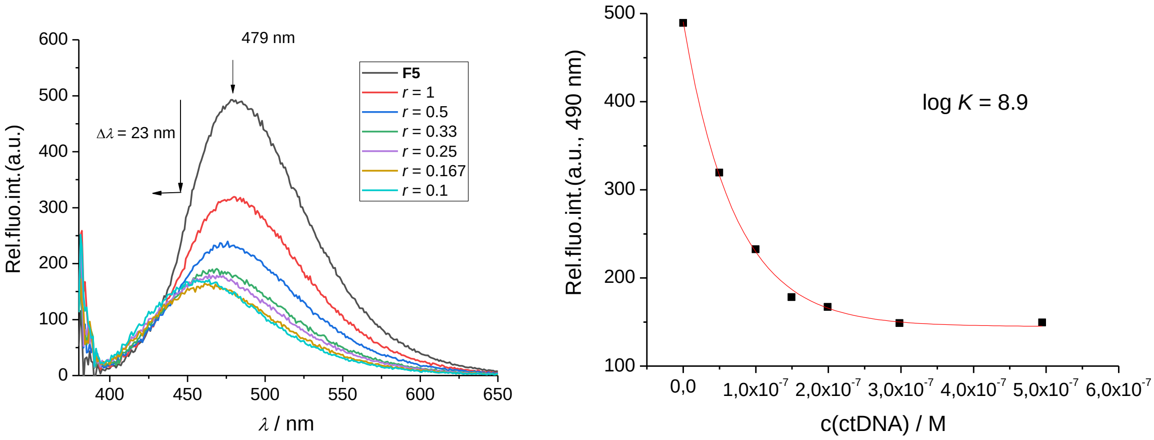

Figure S41. a) Changes in fluorescence spectrum of **F5** ($c = 5 \times 10^{-8}$ M, λ_{exc}= 366 nm) upon titration with **ctDNA** ($c = 5 \times 10^{-8} – 5 \times 10^{-7}$ M), slit: 20-20; b) Dependence of **F5** emission at λ_{max} = 479 nm on c(ctDNA), at pH 7.0, sodium cacodylate buffer, I = 0.05 M.

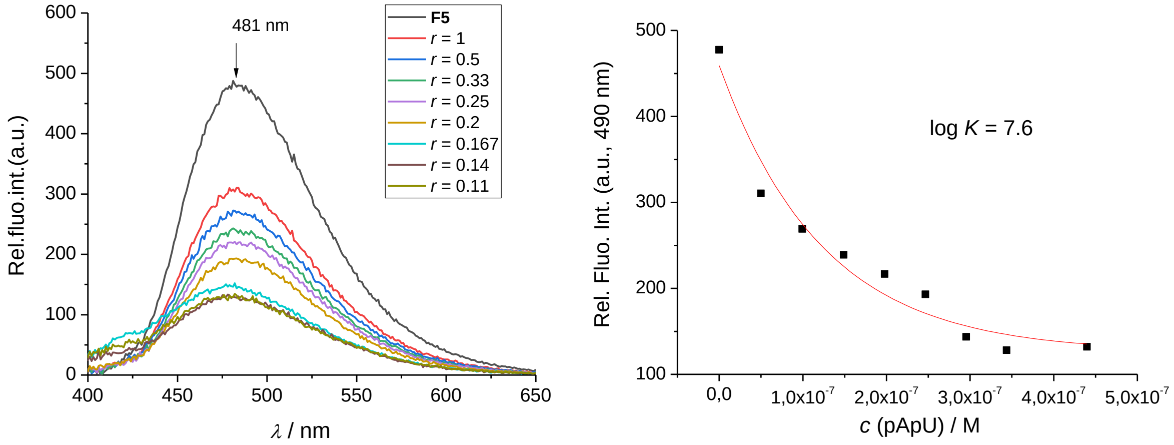

Figure S42. a) Changes in fluorescence spectrum of **F5** ($c = 5 \times 10^{-8}$ M, λ_{exc}= 366 nm) upon titration with **pApU** ($c = 5 \times 10^{-8} – 4.4 \times 10^{-7}$ M), slit: 5-10; b) Dependence of **F5** emission at λ_{max} = 481 nm on c(pApU), at pH 7.0, sodium cacodylate buffer, I = 0.05 M.

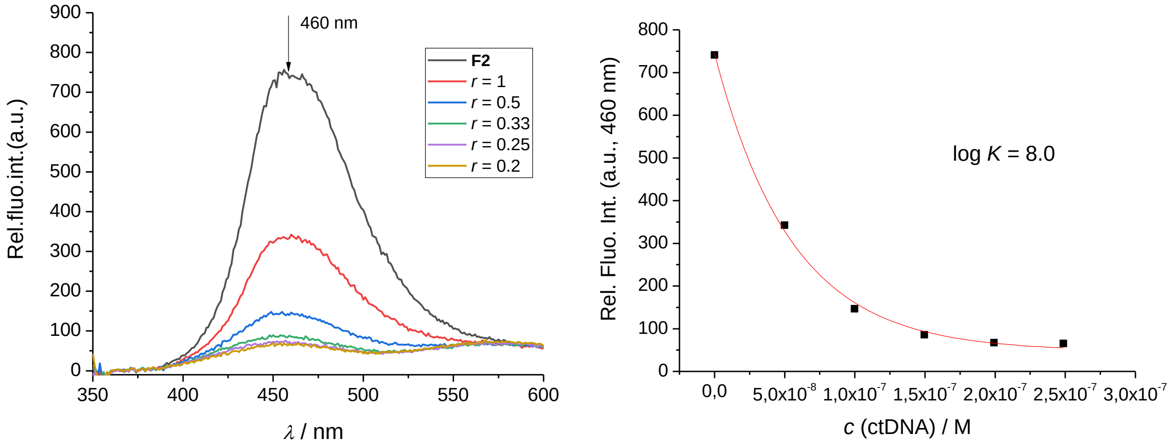

Figure S43. a) Changes in fluorescence spectrum of **F2** ($c = 5 \times 10^{-8}$ M, λ_{exc}= 335 nm) upon titration with **ctDNA** ($c = 5 \times 10^{-8} – 4 \times 10^{-7}$ M), slit: 20-20; b) Dependence of **F2** emission at λ_{max} = 460 nm on c(ctDNA), at pH 7.0, sodium cacodylate buffer, I = 0.05 M.

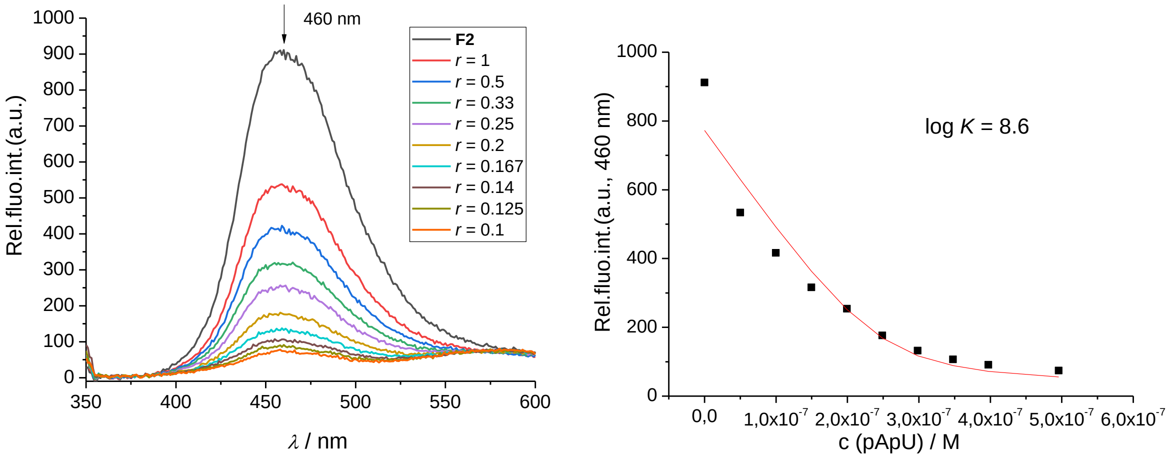

Figure S44. a) Changes in fluorescence spectrum of **F2** ($c = 5 \times 10^{-8}$ M, λ_{exc}= 335 nm) upon titration with **pApU** ($c = 5 \times 10^{-8} – 5 \times 10^{-7}$ M), slit: 20-20 b) Dependence of **F2** emission at λ_{max} = 460 nm on c(pApU), at pH 7.0, sodium cacodylate buffer, I = 0.05 M.

2.3. Circular dichroism (CD) experiments

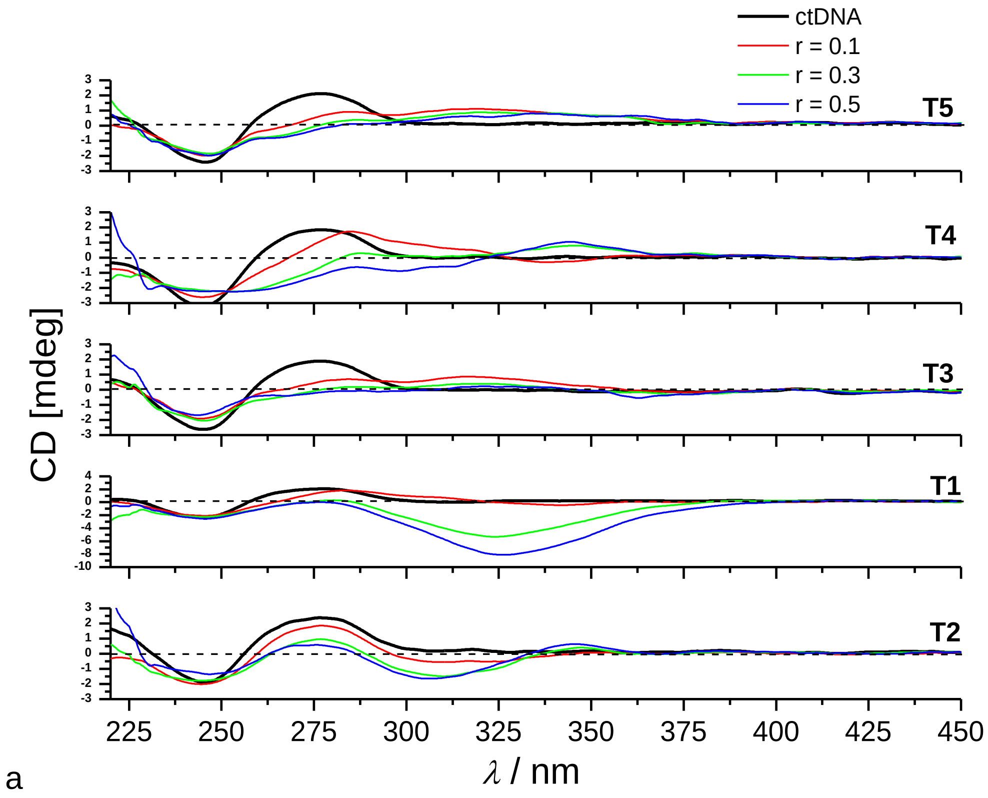

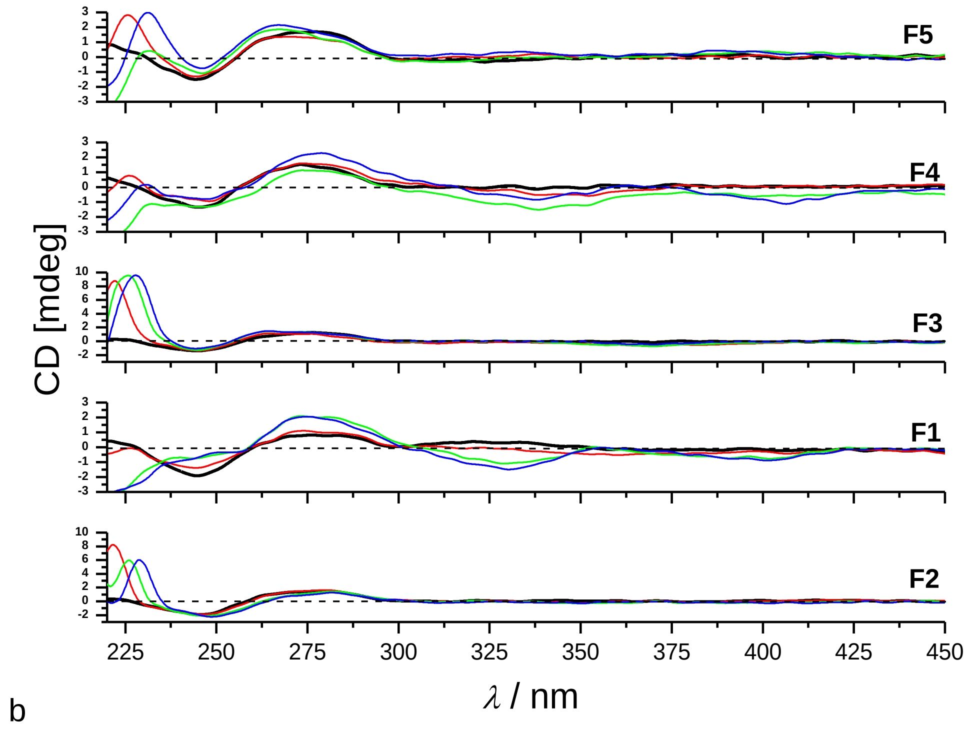

Figure S45. CD titration of ctDNA with: a) **T1**-**T5** (c(polynucleotide) = 3 × 10^{-5} M), and b) **F1**-**F5** (c(polynucleotide) = 2 × 10^{-5} M) at molar ratios r = [compound] / [polynucleotide] (pH 7, buffer sodium cacodylate, I = 0.05 M).

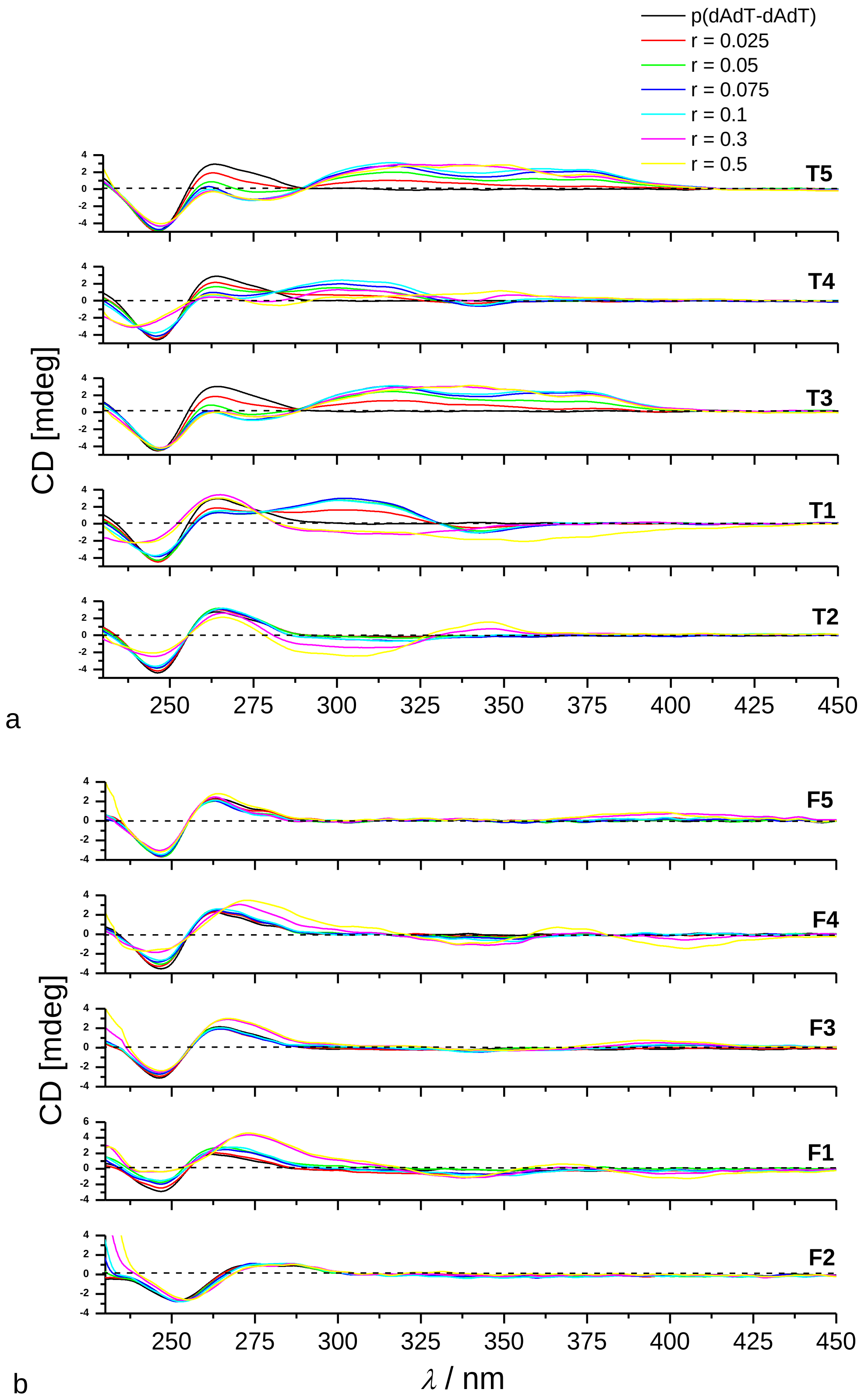

Figure S46. CD titration of p(dAdT)$_2$ with: a) **T1-T5** (*c*(polynucleotide) = 3 × 10^{-5} M), and b) **F1-F5** (*c*(polynucleotide) = 2 × 10^{-5} M) at molar ratios *r* = [compound] / [polynucleotide] (pH 7, buffer sodium cacodylate, *I* = 0.05 M).

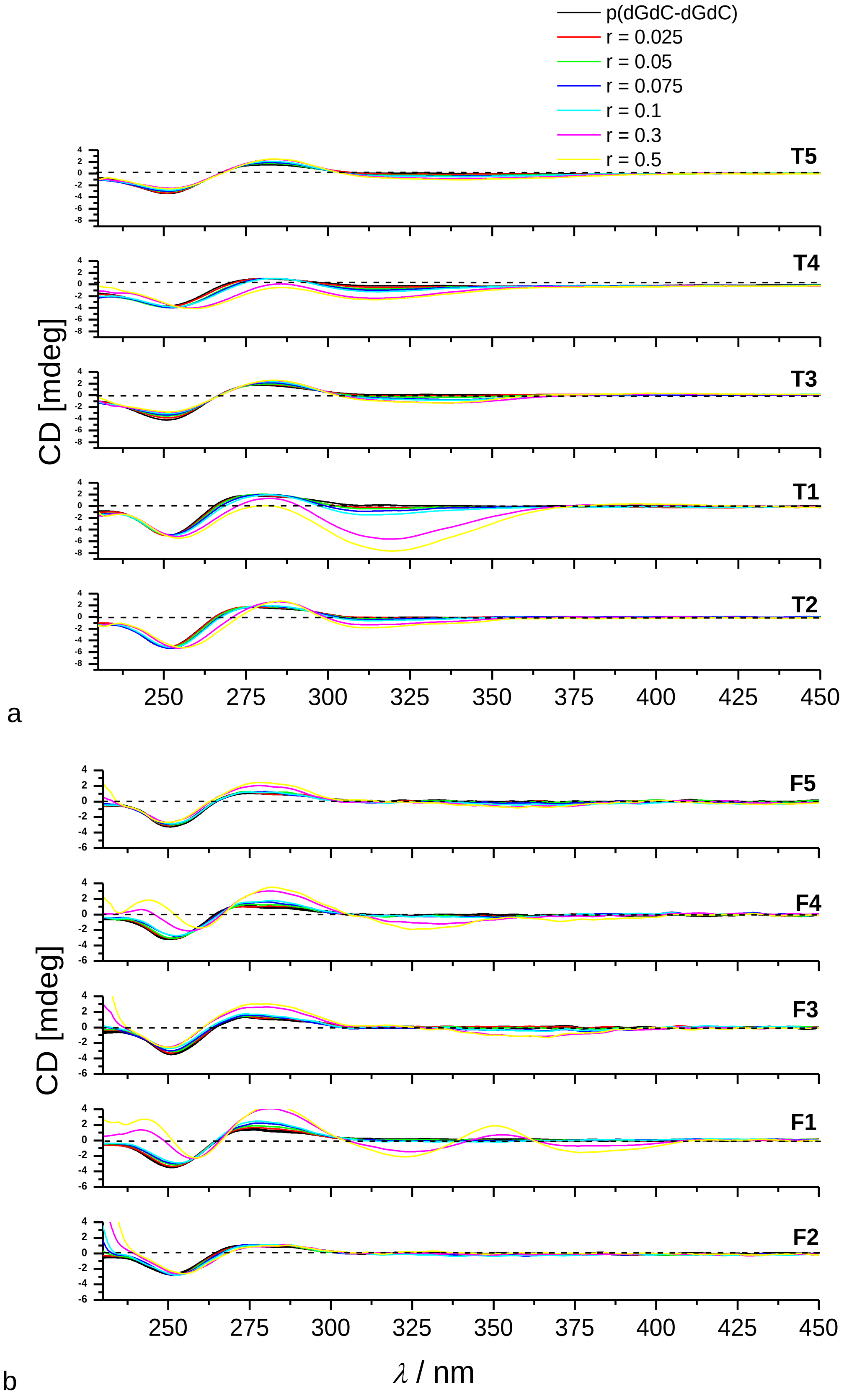

Figure S47. CD titration of p(dGdC)$_2$ with: a) **T1**-**T5** (*c*(polynucleotide) = 3 × 10^{-5} M), and b) **F1**-**F5** (*c*(polynucleotide) = 2 × 10^{-5} M) at molar ratios *r* = [compound] / [polynucleotide] (pH 7, buffer sodium cacodylate, *I* = 0.05 M).

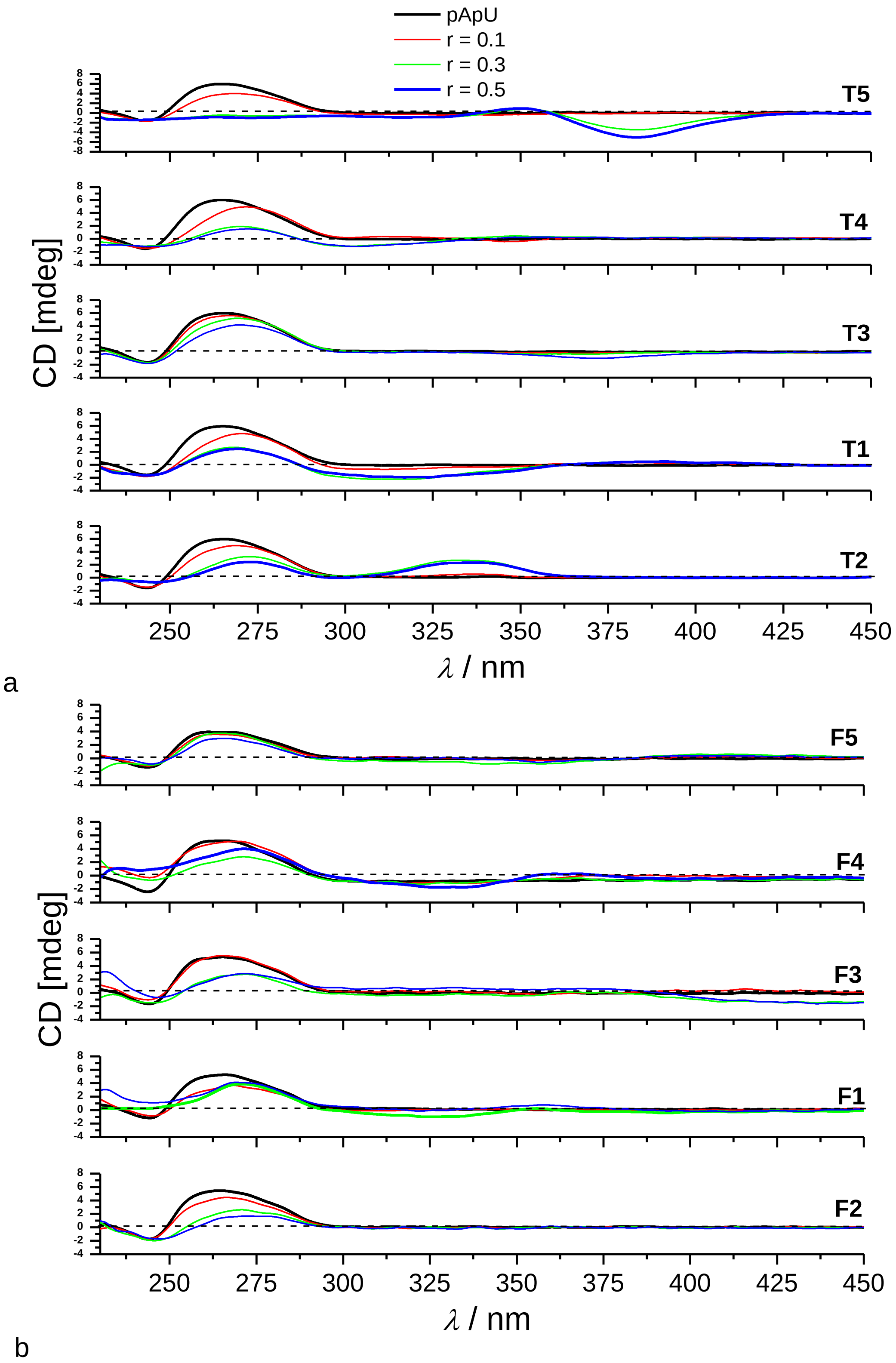

Figure S48. CD titration of pAU with: a) **T1-T5** (c(polynucleotide) = 3×10^{-5} M), and b) **F1-F5** (c(polynucleotide) = 2×10^{-5} M) at molar ratios r = [compound] / [polynucleotide] (pH 7, buffer sodium cacodylate, I = 0.05 M).

3. Synthesis and characterization

All precursors and final compounds **F1**, **T2** and **T3** were previously synthetized and described by our group[3]. The rest of quaternizations are described below.

Compound F2.The quaternization was carried out with iodomethane in DMF, after stirring for 3 days it was poured on diethyl ether and oil appeared. After an ultrasonic bath an orange solid was formed (41%). ^{1}H NMR (500 MHz, DMSO) δ 8.96 (d, *J* = 6.5 Hz, 8H, ArH), 8.55 (s, 2H, ArH), 8.53 – 8.46 (m, 12H, ArH + 4xCH), 7.95 (d, *J* = 16.0 Hz, 4H, 4xCH), 4.33 (s, 12H, 4xCH_3).

Compound F3. The quaternization was carried out with iodomethane in MeOH, after stirring for 5 days a dark orange solid precipitated. It was boiled in CH_2CL_2 (68%) ^{1}H NMR (400 MHz, $CDCl_3$) δ 8.60 (s, 8H, ArH), 7.81 (s, 2H), 7.60 (d, *J* = 16.1 Hz, 4H, 4xCH=), 7.38 (d, *J* = 5.2 Hz, 8H. ArH), 7.01 (d, *J* = 16.0 Hz, 4H, 4xCH=), 1.72 (s, 12H, 4xCH_3).

Compound F4. The quaternization was carried out with propargyl bromide in DMF, after stirring for 2 days it was poured on diethyl ether and a dark orange solid precipitate (60%) ^{1}H NMR (400 MHz, DMSO) δ 8.16 (s, 2H, ArH), 8.06 – 7.88 (m, 20H, ArH), 7.50 (d, *J* = 16.0 Hz, 4H, 4xCH=), 5.04 (s, 8H, 4xCH_2), 3.66 (s, 24H, 8xCH_3), 2.46 (s, 4H, 4xC≡)

Compound F5. The quaternization was carried out with iodobutane in DMF, after stirring for 2 days it was poured on diethyl ether and a dark red solid precipitate (96%) ^{1}H NMR (400 MHz, DMSO) δ 9.06 (d, *J* = 6.5 Hz, 8H, ArH), 8.55 – 8.42 (m, 14H, ArH), 7.94 (d, *J* = 16.0 Hz, 4H, 4xCH=), 4.54 (t, *J* = 7.5 Hz, 8H, 4xCH_2), 1.89 (t, *J* = 7.7 Hz, 8H, 4xCH_2), 1.28 (q, *J* = 7.5 Hz, 8H, 4xCH_2), 0.90 (t, *J* = 7.3 Hz, 12H, 4xCH_3).

Compound T4. The quaternization was carried out with propargyl bromide in DMF, after stirring for 2 days it was poured on diethyl ether and a dark yellow solid precipitate (58%) ^{1}H NMR (400 MHz, DMSO) δ 7.97 (d, *J* = 8.6 Hz, 6H, ArH), 7.86 (d, *J* = 7.8 Hz, 8H, ArH), 7.50 (s, 6H, ArH), 4.98 (s, 6H, 3xCH_2), 3.64 (s, 18H, 6xCH_3), 2.46 (s, 3H, 3xC≡).

Compound T5. The quaternization was carried out with iodobutane in DMF, after sirring for 2 days it was poured on diethyl ether and a light brown precipitate (33%) ^{1}H

NMR (400 MHz, DMSO) δ 9.00 (d, *J* = 6.6 Hz, 6H, ArH), 8.30 (d, *J* = 6.5 Hz, 6H, ArH), 8.18 (s, 3H, ArH), 8.10 (d, *J* = 16.3 Hz, 3H, 3xCH_2), 7.78 (d, *J* = 16.3 Hz, 3H, 3xCH_2), 4.51 (t, *J* = 7.4 Hz, 6H, 3xCH_2), 1.97 – 1.76 (m, 6H, 3xCH_2), 1.35 – 1.20 (m, 7H, 3xCH_2), 0.89 (t, *J* = 7.4 Hz, 9H,3xCH_3).

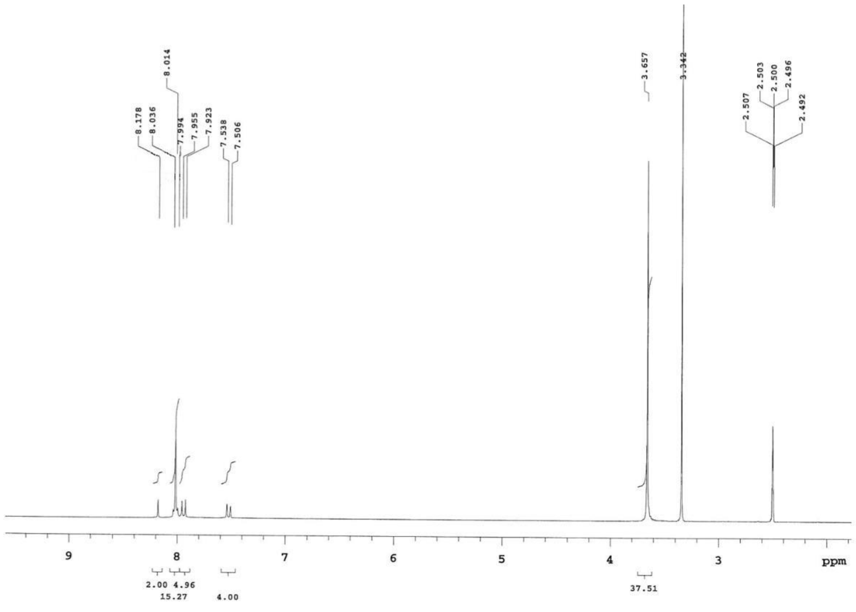

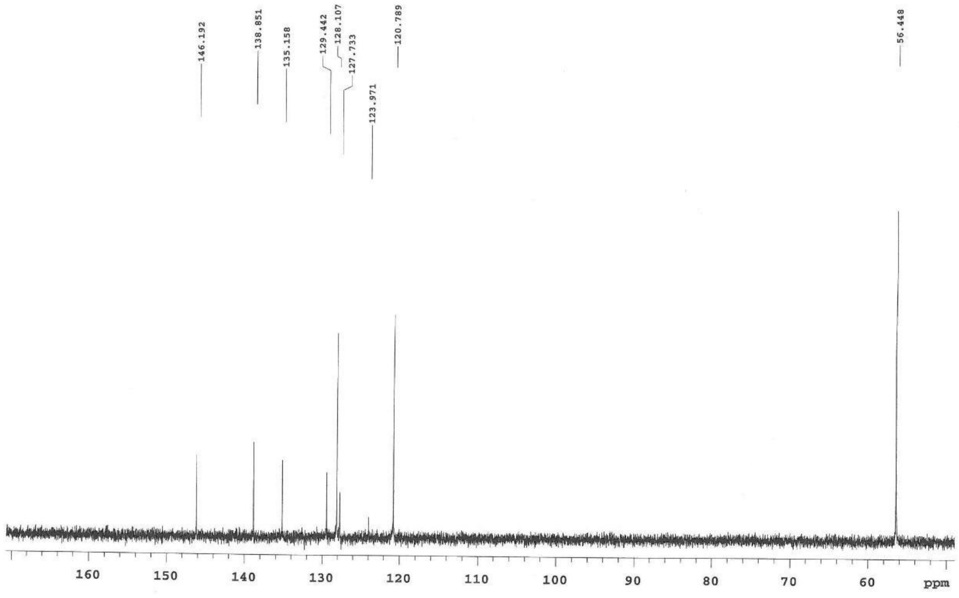

Figure S49. 1H NMR and 13C NMR of compound **2**; key precursor of **F1**.

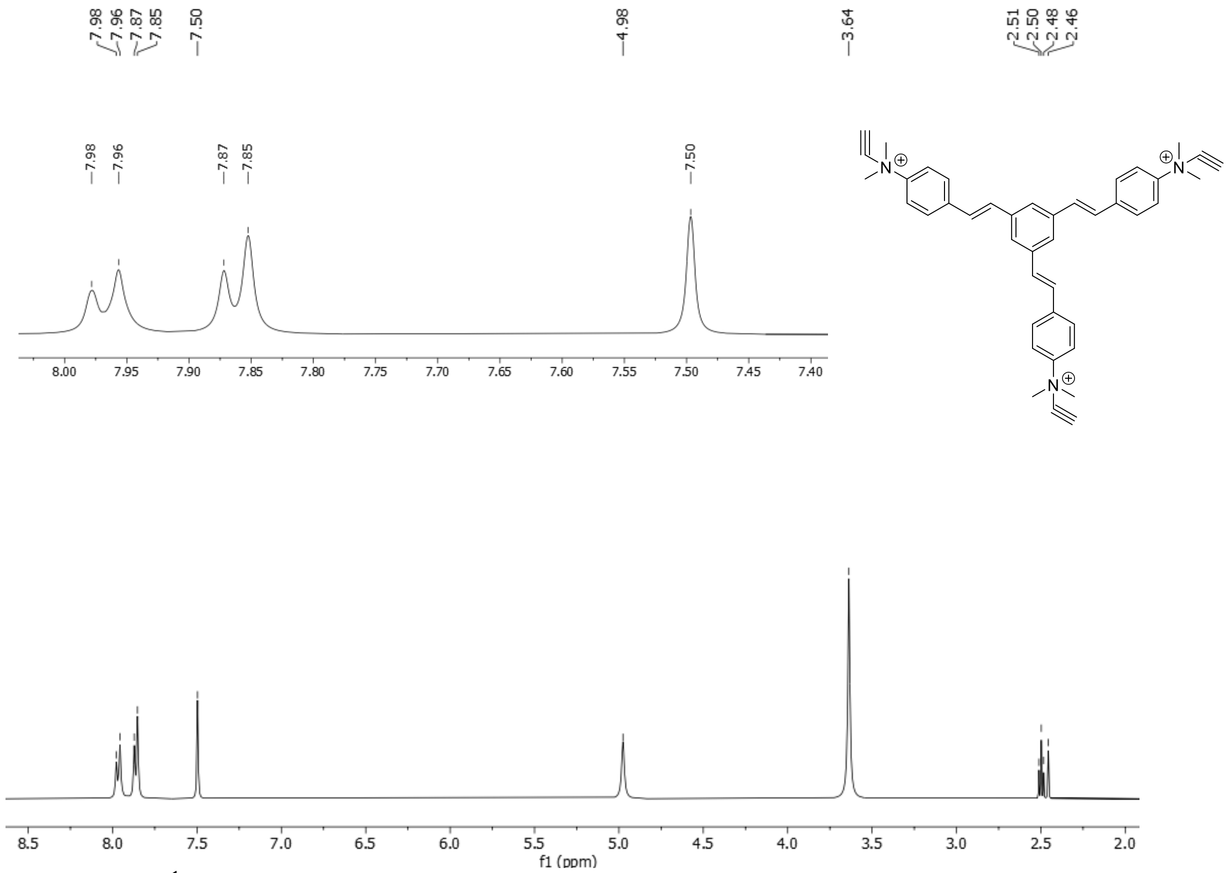

Figure S50. ^{1}H-NMR of compound **T4**

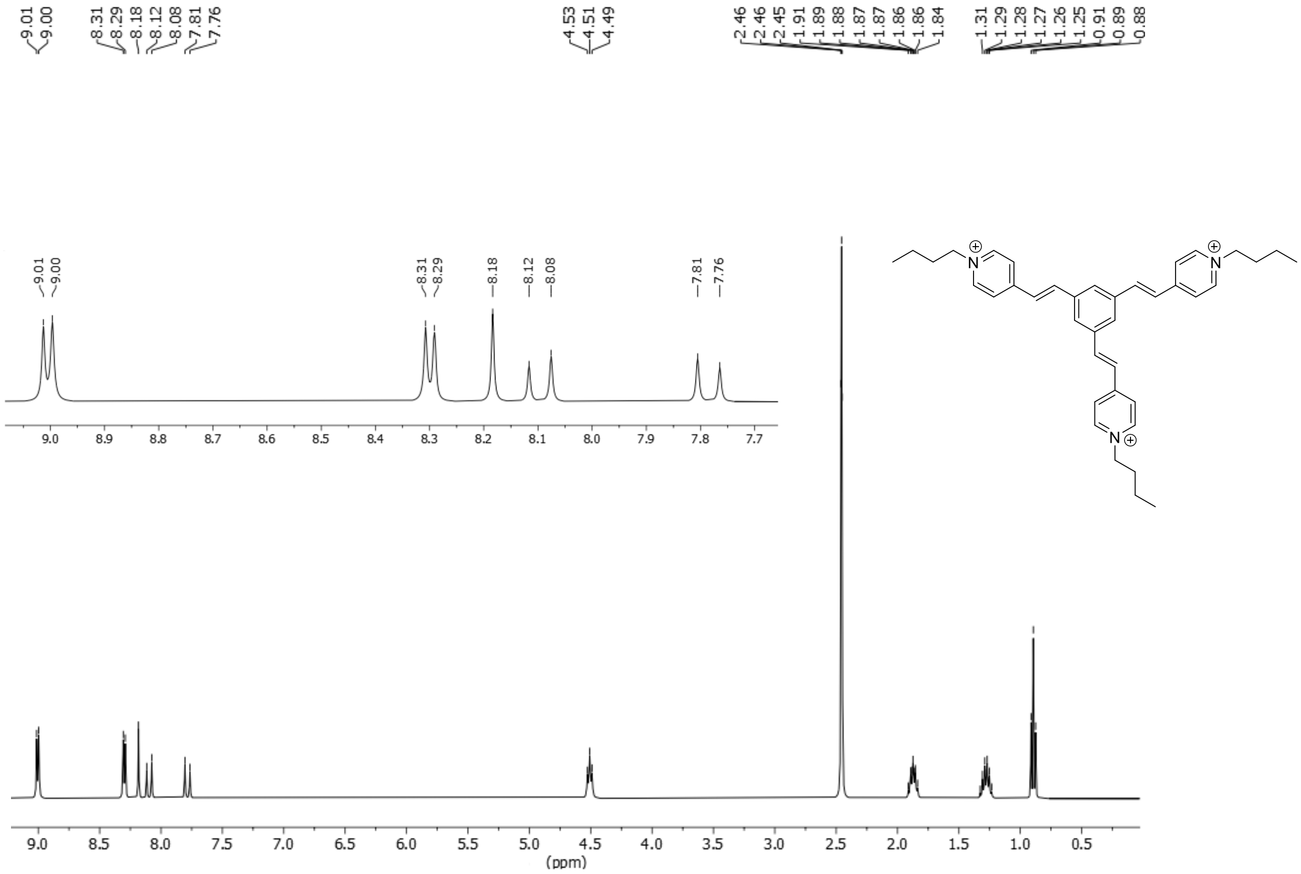

Figure S51. ^{1}H-NMR of compound **T5**

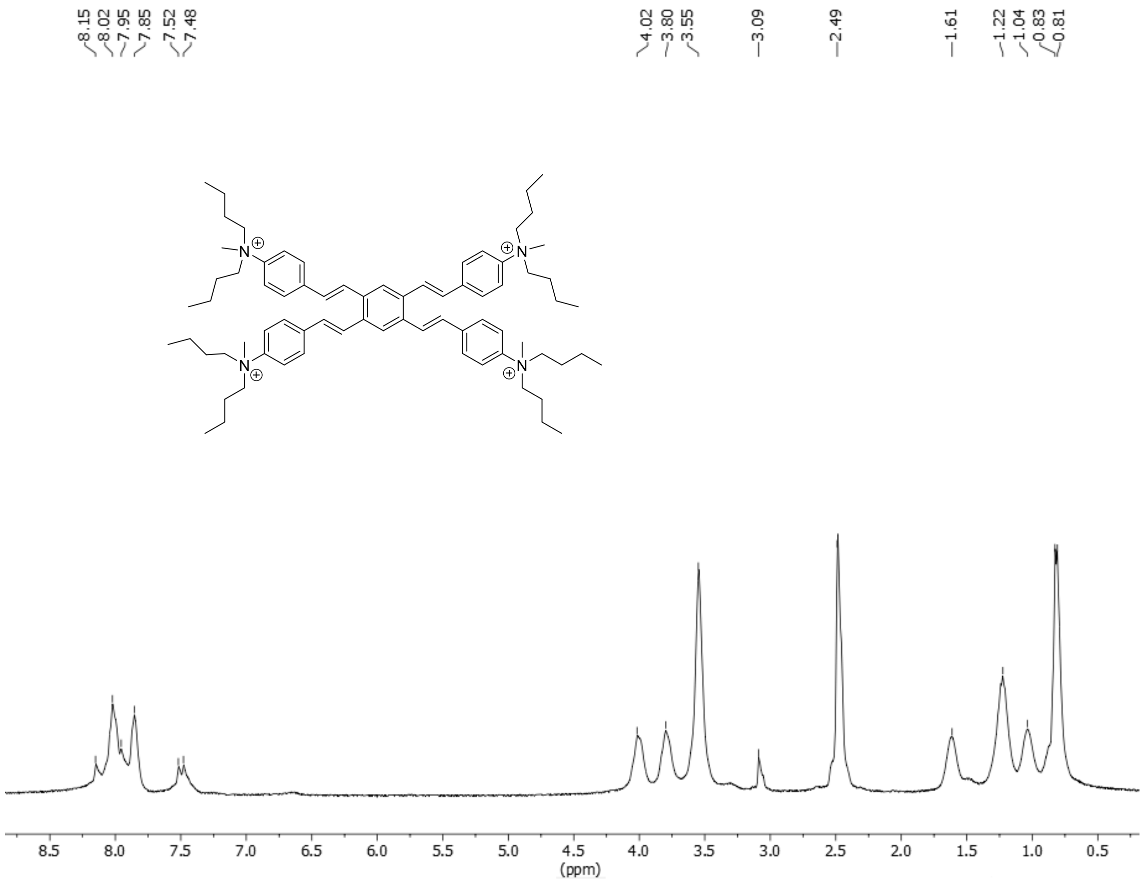

Figure S52. ^{1}H-NMR of compound **F2**

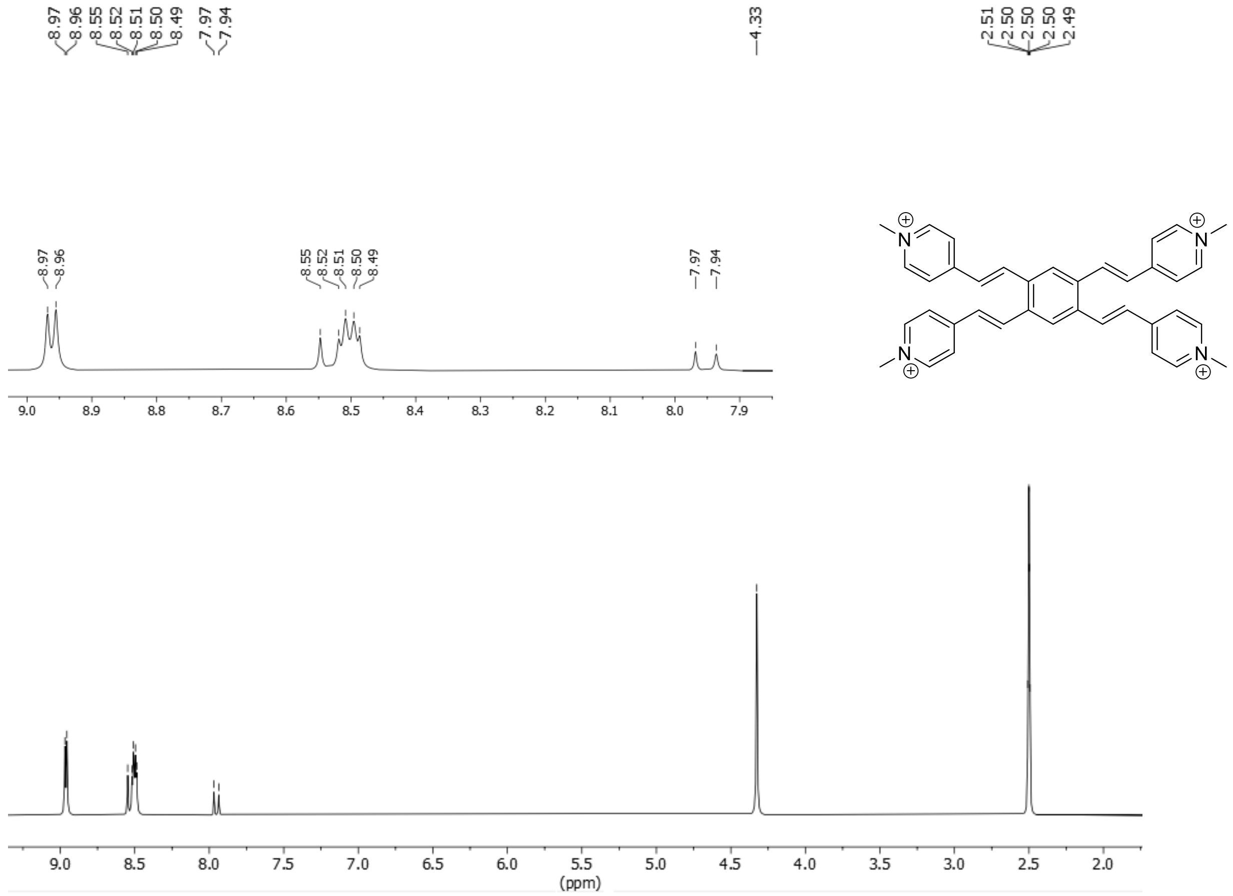

Figure S53. ^{1}H-NMR of compound **F3**

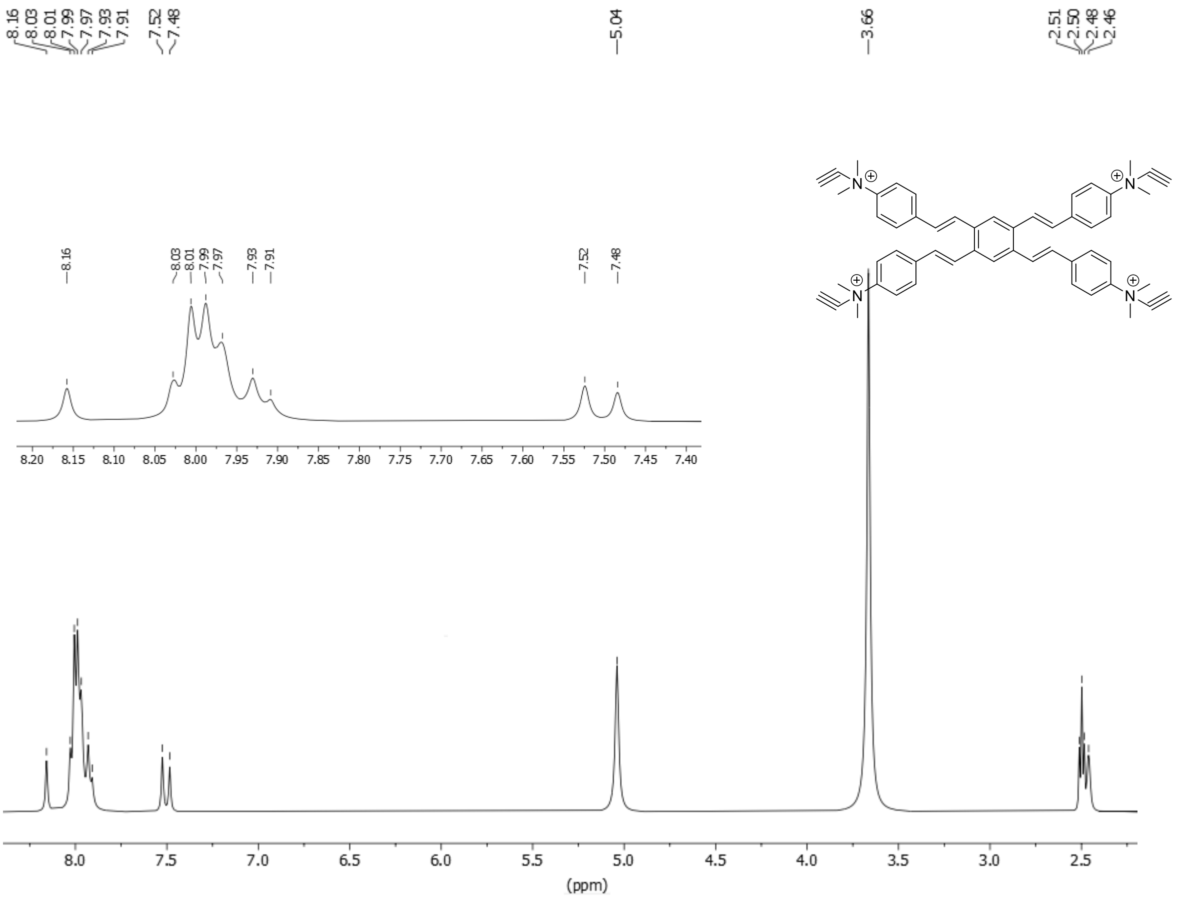

Figure S54. ^{1}H-NMR of compound **F4**

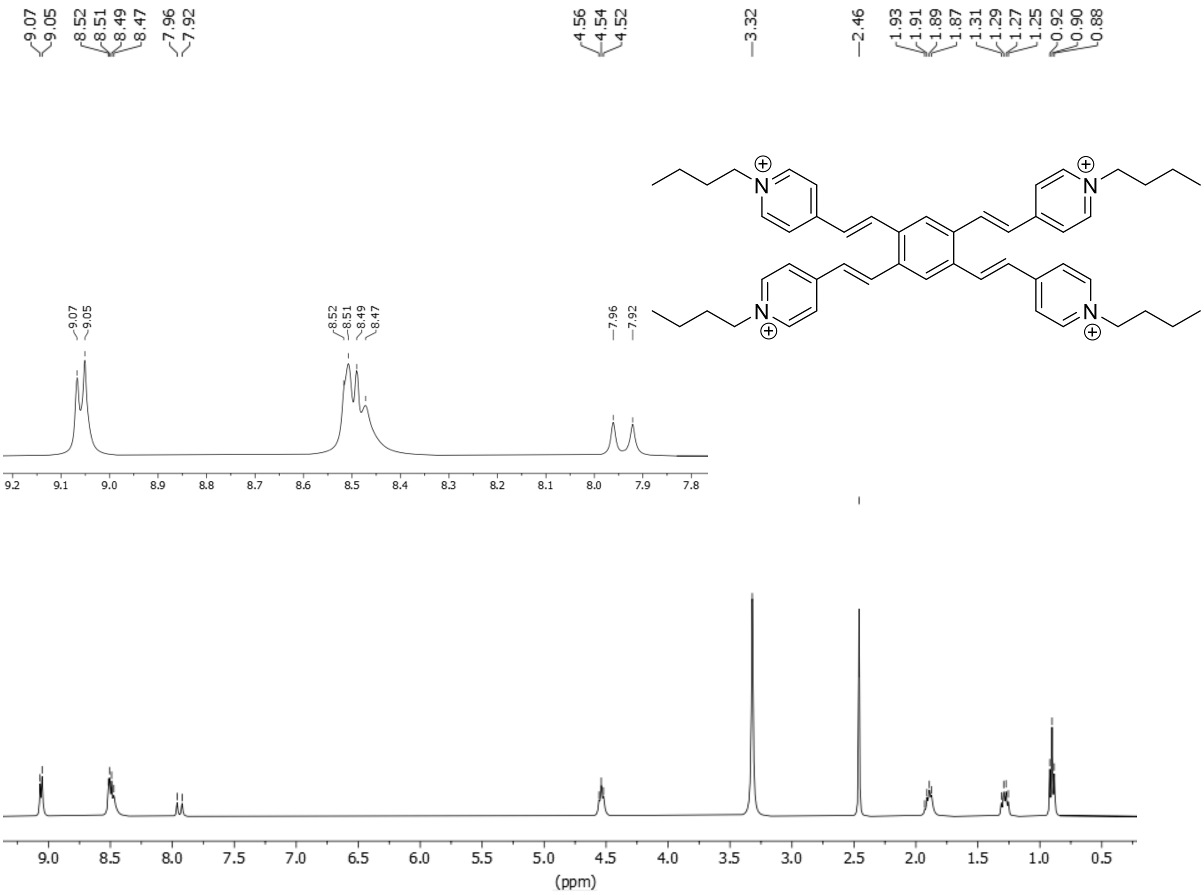

Figure S55. ^{1}H-NMR of compound **F5**

References

[1] Saenger, W. Principles of Nucleic Acid Structure; Springer-Verlag: New York, USA, 1983.

[2] Cantor, C.R.; Schimmel; P.R. Biophysical Chemistry, WH Freeman and Co.: San Francisco, USA, 1980; pp. 1109-1181.

[3] Tolosa, J., de las Heras, G. S., Carrión, B., Segura, T., Páez, P. L., de Lera-Garrido, F. J., Rodríguez-López, J., and García-Martínez, J. C. Structure-Activity Relationships for Poly(phenylene)vinylene Derivatives as Antibacterial Agents, Chemistryselect **2018**, *3*, 7327-7332. doi: 10.1002/slct.201801287
